# IMGT^®^ *Homo sapiens* IG and TR Loci, Gene Order, CNV and Haplotypes: New Concepts as a Paradigm for Jawed Vertebrates Genome Assemblies

**DOI:** 10.3390/biom12030381

**Published:** 2022-02-28

**Authors:** Marie-Paule Lefranc, Gérard Lefranc

**Affiliations:** IMGT®, The International ImMunoGeneTics Information System®, Laboratoire d’Immuno Génétique Moléculaire (LIGM), Institut de Génétique Humaine (IGH), Université de Montpellier (UM), Centre National de la Recherche Scientifique (CNRS), UMR 9002 CNRS-UM, 141 rue de la Cardonille, CEDEX 5, 34396 Montpellier, France

**Keywords:** IMGT, immunogenetics, immunoinformatics, immunoglobulin (IG), antibody, T cell receptor (TR), system biology, bioengineering, copy number variation (CNV), haplotype

## Abstract

IMGT^®^, the international ImMunoGeneTics information system^®^, created in 1989, by Marie-Paule Lefranc (Université de Montpellier and CNRS), marked the advent of immunoinformatics, a new science which emerged at the interface between immunogenetics and bioinformatics for the study of the adaptive immune responses. IMGT^®^ is based on a standardized nomenclature of the immunoglobulin (IG) and T cell receptor (TR) genes and alleles from fish to humans and on the IMGT unique numbering for the variable (V) and constant (C) domains of the immunoglobulin superfamily (IgSF) of vertebrates and invertebrates, and for the groove (G) domain of the major histocompatibility (MH) and MH superfamily (MhSF) proteins. IMGT^®^ comprises 7 databases, 17 tools and more than 25,000 pages of web resources for sequences, genes and structures, based on the IMGT Scientific chart rules generated from the IMGT-ONTOLOGY axioms and concepts. IMGT^®^ reference directories are used for the analysis of the NGS high-throughput expressed IG and TR repertoires (natural, synthetic and/or bioengineered) and for bridging sequences, two-dimensional (2D) and three-dimensional (3D) structures. This manuscript focuses on the IMGT^®^ *Homo sapiens* IG and TR loci, gene order, copy number variation (CNV) and haplotypes new concepts, as a paradigm for jawed vertebrates genome assemblies.

## 1. Introduction

The adaptive immune response of the jawed vertebrates (or *gnathostomata*), which appeared in evolution about 450 million years ago is characterized by a remarkable immune specificity and memory, which are the properties of the B and T cells owing to an extreme diversity of their antigen receptors, the immunoglobulins (IG) or antibodies and the T cell receptors (TR), respectively [1]. In humans and other mammals, an IG consists of two identical light chains (kappa (IGK) or lambda (IGL)) and two identical heavy chains (IGH) [2], while a TR consists of two chains, either alpha (TRA) and beta (TRB), or gamma (TRG) and delta (TRD) [3]. Each IG and TR chain comprises a variable domain (V-DOMAIN) which determines the specificity for the antigen, and a constant region (C-REGION) composed of one, three or four constant domains (C-DOMAIN) depending on the chain type [4,5]. The V-DOMAIN results from the genomic rearrangement of variable (V), diversity (D) and joining (J) genes for IGH, TRB and TRD chains (encoding a V-D-J-REGION) and of V and J genes for IGK, IGL, TRA and TRG chains (encoding a V-J-REGION) [1,2,3,4,5]. Additional mechanisms occurring during the rearrangements (N diversity, somatic hypermutations for the IG [5]) contribute to the extreme diversity of the IG and TR (theoretically 10^12^ IG and TR per individual, which is only limited by the number of the B and T cells that an organism is genetically programmed to produce) [1].

**Figure 1:**
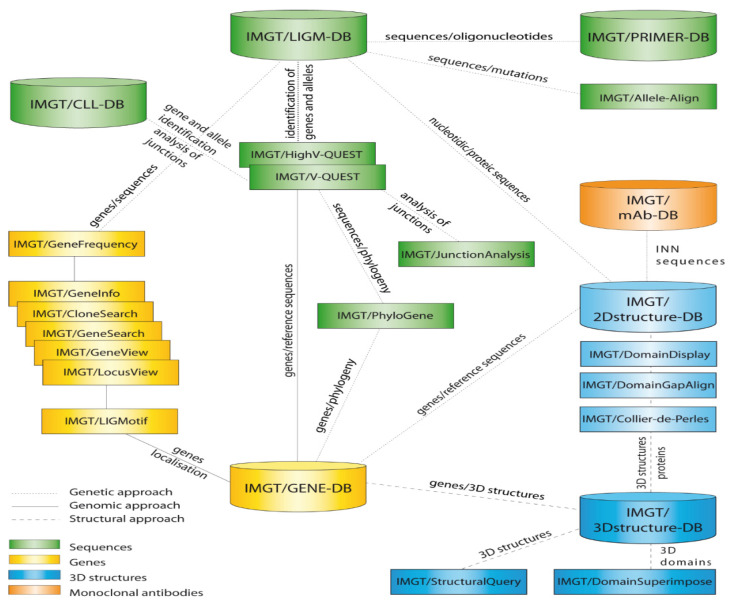
IMGT^®^, the international ImMunoGenetics information system^®^, http://www.imgt.org (accessed on 22 February 2022) [1,5]. IMGT^®^ comprises seven IMGT databases (shown as cylinders), seventeen online IMGT tools (shown as rectangles) and the IMGT Web resources (more than 25,000 pages, the ‘IMGT Marie-Paule page’) (not shown), for genes (in yellow), sequences (in green) and structures (in blue), all available from the IMGT^®^ Home page. IMGT/mAb-DB has been online since 4 December 2009. IMGT/HighV-QUEST for next-generation sequencing (NGS) high-throughput sequence analysis, created in October 2010, has been available on the web since 22 November 2010. (With permission from M-P. Lefranc and G. Lefranc, LIGM, Founders and Authors of IMGT^®^, the international ImMunoGeneTics information system^®^, http://www.imgt.org) (accessed on 22 February 2022).

IMGT^®^, the international ImMunoGeneTics information system^®^ (http://www.imgt.org) (accessed on 22 February 2022) (Figure 1) [1,2,3,4,5], was created in 1989 by Marie-Paule Lefranc (Université de Montpellier and CNRS) in order to characterize the genes and alleles involved in the IG and TR synthesis of vertebrate species from fish to human, and to standardize and manage the huge and complex diversity of IG and TR sequences and structures. The founding of IMGT^®^ marked the birth of immunoinformatics [1], a new science, which emerged at the interface between immunogenetics and bioinformatics. For the first time, IG and TR genes (V, D, J and C) were officially recognized as “genes” as well as were the conventional genes [2,3,6,7]. This major breakthrough allowed genes and data of the complex and highly diversified adaptive immune responses to be managed in genomic databases and tools [1]. IMGT^®^, the international ImMunoGenetics information system^®^ has been online since 1995 (the first Internet connexion of IMGT/LIGM-DB occurred at the 9th International Congress of Immunology (ICI) in San Francisco, CA, USA), 23–29 July 1995), marking the 7-year anniversary of the first Internet France-USA connexion of 28 July 1988). The IG and TR nomenclature [1,2,3,4,5,6,7], based on the internationally acknowledged expertise of the Laboratoire d’ImmunoGénétique Moléculaire (LIGM), has been endorsed since 1992 by the World Health Organization—International Union of Immunological Societies (WHO-IUIS) [8,9], making IMGT^®^, the global reference in immunogenetics and immunoinformatics. 

IMGT^®^ is an integrated knowledge system for sequences, genes and structures of the IG or antibodies, TR and major histocompatibility (MH) proteins of the adaptive immune responses of the jawed vertebrates, as well as of other proteins of the IG superfamily (IgSF) and MH superfamily (MhSF) of vertebrates and invertebrates and of related proteins of immunological interest (RPI) [1,2,3,4,5]. The strength of the system is to have been built on the scientific rules (standardized keywords, standardized labels, standardized gene and allele names, a unique numbering for domains) elaborated by LIGM, with the goal to bridge genes, nucleotide and amino acid sequences and structures for analyzing their diversity and understanding biological functions. The first nucleotide sequences database for the genes of the adaptive immune responses in immunology, LIGM-DB/IMGT [10,11], became rapidly international [12,13,14], with a systematized approach for data coherence and distribution [15,16,17,18,19,20] and the building of an information system for comparative immunogenetics and immunology [21,22,23]. In 2003, IMGT was acknowledged as the global reference in immunoinformatics [24,25] with high-quality databases, tools and web resources, publicly available for IG and TR sequence analysis and antibody engineering [26]. At the research level, it was the demonstration that an ontology (formalization of the LIGM standardized rules in IMGT-ONTOLOGY [27,28,29]) and a system (implementation of IMGT-Choreography for genomics, genetics and structural approaches [30,31,32,33,34,35,36,37,38]) have bridged biological and computational spheres in bioinformatics [39,40]. As examples, IMGT^®^ databases, tools and web resources are used for genome diversity and evolution studies, immune repertoire analysis, in medical research (repertoire in autoimmune diseases, AIDS, leukemia and lymphoma) and therapeutic antibody engineering and humanization [23,41,42,43,44,45,46,47,48,49,50,51].

IMGT^®^ comprises seven IMGT databases, seventeen online IMGT tools (Figure 1) and the IMGT Web resources (more than 25,000 pages, the ‘IMGT Marie-Paule page’), all available from the IMGT Home page (http://www.imgt.org) (accessed on 22 February 2022) [1,2,3,4,5]. IMGT^®^ databases are specialized in sequences (IMGT/LIGM-DB [52], IMGT/PRIMER-DB [53,54], IMGT/CLL-DB [55]), genes and alleles (IMGT/GENE-DB [56]), two-dimensional (2D) and three-dimensional (3D) structures (IMGT/2Dstructure-DB, IMGT/3Dstructure-DB) [57,58,59]. IMGT/2Dstructure-DB and IMGT/3Dstructure-DB use the same computing frame and provide gene and allele identification and molecular characterization of the amino sequences of the IG and TR receptors, chains and domains [1,2,3,4,5,60]. In addition, IMGT/3Dstructure-DB provides contact analysis between domains, standardized pMH contact sites, and amino acid characterization of paratope [61] and epitope [62] in IG/Ligand and TR/peptide/MH complexes [63,64]. IMGT/mAb-DB [1,65], the IMGT database and interface for therapeutic adaptive immune responses proteins (IG such as monoclonal antibodies (mAb), antibody–drug conjugates (ADC) or chimeric antigen receptors (CAR T), and TR), fusion proteins for immune applications (FPIA) and composite proteins for clinical applications (CPCA), has been online since 4 December 2009. IMGT/mAb-DB has reciprocal links with the IMGT/2Dstructure-DB (and IMGT/3Dstructure-DB, if 3D structures are available) and allows query on the International Nonproprietary Names (INN) of the World Health Organization (WHO) [66,67]. It also provides links to the lists and definitions published twice a year by the WHO INN programme and to the USA Food and Drug Administration (FDA) approvals. 

IMGT^®^ tools for nucleotide sequence analysis comprise IMGT/V-QUEST [68,69,70,71,72,73] which implements IMGT/JunctionAnalysis [74,75,76,77] and IMGT/Automat [78,79], and IMGT/HighV-QUEST [73,80,81,82,83], its high-throughput version for next-generation sequencing (NGS). Created in October 2010 and available on the web since 22 November 2010, IMGT/HighV-QUEST uses the same algorithm and IMGT reference directories and provides the same functionalities as IMGT/V-QUEST. It includes, as an option, a statistical module for the characterization of IMGT (AA) clonotypes [82]. Pairwise comparisons of the diversity and expression of the IMGT (AA) clonotypes can be performed using IMGT/StatClonotype [84,85], a package downloadable from the website. IMGT/DomainGapAlign [58,86,87] is the IMGT^®^ tool for amino acid sequence analysis widely used for domain antibody engineering and humanization. IMGT tools for genomic analysis include IMGT/LIGMotif [88] used for genomic annotations and a set of tools for visualization and analysis, IMGT/LocusView, IMGT/GeneView, IMGT/GeneSearch, IMGT/CloneSearch, IMGT/GeneFrequency, IMGT/AlleleAlign, IMGT/PhyloGene [89], IMGT/GeneInfo [90,91]. The IMGT^®^ Web resources (the ‘IMGT Marie-Paule page’) comprise IMGT Repertoire (IG and TR, MH, and RPI), IMGT Scientific chart, IMGT Index, IMGT Bloc-notes, IMGT Education, IMGT Posters and diaporama, The IMGT Medical page, The IMGT Veterinary page, The IMGT Biotechnology page (Antibody engineering) and The IMGT Immunoinformatics page [1].

The accuracy and the consistency of the IMGT data through the IMGT information system [1,2,3,4,5] are based on IMGT-ONTOLOGY [92,93,94,95,96,97,98,99,100,101,102,103,104,105,106,107,108,109,110,111,112,113], the first ontology for immunogenetics and immunoinformatics, and on the IMGT Scientific chart rules generated from its axioms and concepts [1]. They include the controlled vocabulary and annotation rules that comprise the IMGT^®^ standardized keywords (IDENTIFICATION axiom) [96], the IMGT^®^ standardized labels (DESCRIPTION axiom) [97], the IMGT^®^ gene and allele nomenclature (CLASSIFICATION axiom) [98], the IMGT^®^ unique numbering [99,100,101,102,103,104,105,106] for variable V [101], constant C [102] and groove G [103] domains, and IMGT^®^ Colliers de Perles [107,108,109,110,111,112] (NUMEROTATION axiom), with standardized IMGT physicochemical classes of the 20 common amino acids [113]. 

Links to biocurated data of IG and TR genes and alleles from fish to humans are provided in IMGT^®^ Creations and updates (http://www.imgt.org/IMGTinformation/creations/) (access in ‘References and News’ on the IMGT^®^ Home page) (accessed on 22 February 2022), online since October 1998. IG and TR nomenclature (genes and alleles names) and IMGT unique numbering of the IMGT^®^ Creations and updates are validated by the International Union of Immunological Societies (IUIS) Nomenclature Committee (NOM) Immunoglobulins (IG), T cell receptors (TR) and major histocompatibility (MH) Sub-Committee (IMGT-NC) [114].

A primary axis of research and development in immunogenetics and immunoinformatics consists in deciphering the genomic organization of the IG and TR loci in jawed vertebrates. Indeed, new IMGT genes and alleles, described with the IMGT nomenclature, IMGT unique numbering and IMGT standardized keywords and labels [1], enrich the IMGT^®^ databases, tool reference directories and web resources for a better knowledge of natural IG and TR repertoires. These data are used for the analysis of the next generation sequencing (NGS) high-throughput expressed IG and TR repertoires (natural, synthetic and/or bioengineered) and for bridging sequences and two-dimensional (2D) and three-dimensional (3D) structures. This manuscript focuses on the *Homo sapiens* IG and TR loci, gene order, CNV and haplotypes new concepts, as a paradigm for jawed vertebrates genome assemblies. 

## 2. IG and TR Genes and Alleles Paradigm

### 2.1. Advent of Immunoinformatics

The V, D, J, and C genes which code the antigen receptors were officially recognized as ‘genes’, as were the conventional genes, at the 10th Human Genome Mapping (HGM10) Workshop, in New Haven in 1989, marking the creation of IMGT^®^ and the advent of immunoinformatics [1]. The *Homo sapiens* TRG locus [115] was the first complete antigen receptor locus officially approved in 1989 for entry in the HGM database [116,117]. In 1992, the *Homo sapiens* IMGT genes and alleles names were endorsed by the WHO—IUIS NOM (https://iuis.org/committees/nom/) (accessed on 22 February 2022). IMGT-NC became the first IUIS Nomenclature Sub-Committee for immunoglobulins and T cell receptors [114] in charge of providing the IG and TR genes and alleles names and promoting IMGT standards [118,119,120,121]. The human (*Homo sapiens*) IG and TR IMGT gene names [6,7] were approved by the Human Genome Organization (HUGO) Nomenclature Committee (HGNC) in 1999 [122] and entered in the NCBI gene database (first LocusLink, then EntrezGene, superseded by Gene). 

### 2.2. Homo sapiens Locus, Genes and Alleles

The IG and TR genes are classified in groups defined by the gene type (V, D, J or C) and by the locus to which they belong [1,2,3,4,5,6,7,8,9]. The seven major loci, three for IG (IGH, IGK and IGL) [2,4,5] and four for TR (TRA, TRB, TRD and TRG) [3], are located on different chromosomes, with the particularity of the TRD locus being nestled inside the TRA locus in higher vertebrates [3]. The princeps LIGM publications on the IG and TR loci, genes and alleles of the seven human (*Homo sapiens*) loci are the two FactsBooks published in 2001 [2,3]. They comprised for the IG, 203 functional and open reading frame (ORF) genes corresponding to 459 alleles, for a total of 837 sequences [2], and for the TR, 168 functional and ORF genes [3]. Entries of the FactsBooks [2,3] provide information on assignment to subgroups and nomenclature, gene definition and functionality, gene location, allelic polymorphism, standardized sequence alignment with protein translation, framework and complementarity determining region (CDR-IMGT) lengths, two-dimensional representations (or Colliers de Perles), IMGT/LIGM-DB and EMBL/GenBank accession numbers, genome database accession numbers (GDB, LocusLink) and key references [2,3]. This information has served as templates for the IMGT Repertoire (IG and TR) sections on the IMGT website, in the IMGT Web resources (the ‘IMGT Marie-Paule page’) (IMGT^®^ http://www.imgt.org (accessed on 22 February 2022), IMGT Repertoire (IG and TR) (1). Locus and genes; (2). Proteins and alleles; (3). 2D and 3D structures; (4). Probes and RFLP; (5). Taxonomy; (6). Gene regulation and expression; (7). Genes and clinical entities). Basic IMGT Web resources include Gene tables, Alignment of alleles, Protein displays, Colliers de Perles, Locus representations, Potential germline repertoire with CDR-IMGT lengths, Locus gene order, copy number variations (CNV) and haplotypes) [5]. 

This detailed identification, description and classification of the human IG and TR loci, genes and alleles [2,3], using the IMGT Scientific chart rules, is the result of a huge work of annotation and expert analysis, by LIGM, of tens of thousands of nucleotide sequences from phages, cosmids or contigs submitted by the authors to the generalist nucleotide databases (EMBL database, now European Nucleotide Archive (ENA) [123], GenBank [124] and DNA Databank of Japan (DDBJ) [125]. The annotated sequences were integrated into the newly created IMGT/LIGM-DB [10,11], using the EMBL/GenBank/DDBJ accession numbers in order to facilitate interoperability with the generalist nucleotide databases. The *Nature* and *Science* papers on the human genome sequencing [126,127], published in 2001, contain limited information on the genes of the adaptive immune responses. However a careful analysis of the maps published in these papers allowed us to confirm the chromosomal localizations of the seven main loci: IGH, IGK and IGH (for the immunoglobulins), TRA, TRB, TRG and TRD (for the T cell receptors), described in 2001, in the Immunoglobulin FactsBook [2] and T cell receptor FactsBook [3], respectively, and determined by an analysis of translocations involving the IG and/or TR loci in leukemia and lymphoma (http://www.imgt.org/IMGTrepertoire/GenesClinical/translocation/human/overview/Hu_overviewpart1.html) (accessed on 22 February 2022). 

### 2.3. Extension to Mus musculus and Fish (Chondrichtyes and Teleostei)

Based on this paradigm of the human loci (IMGT nomenclature, IMGT unique numbering, IMGT standardized keywords and labels), the seven mouse (*Mus musculus)* loci with a total of 625 genes (377 IG and 248 TR) [128,129,130,131] were characterized and presented at the 19th International Mouse Genome Conference (IMGC) in 2005 ((http://www.imgt.org/IMGTposters/IMGC_IG.html) (accessed on 22 February 2022) and (http://www.imgt.org/IMGTposters/IMGC_TR.html) (accessed on 22 February 2022)), and entered in NCBI Gene, with reciprocal links to IMGT/GENE-DB and in Mouse Genome Informatics (MGI). 

The analysis of IG genes of four Chondrichthyes and twenty-two Teleostei different species confirmed that the IG and TR paradigm was applicable for fish, however, most sequences were at that time unmapped and were assigned a provisional nomenclature with the letter S [132,133]. The Chondrichthyes and Teleostei light chain which is neither kappa nor lambda was defined as ‘iota’ encoded by genes of the IG iota (IGI) locus which includes IGIV, IGIJ and IGIC groups (http://www.imgt.org/IMGTrepertoire/LocusGenes/genetable/Teleostei/#IGIV) (accessed on 22 February 2022).

### 2.4. Homo sapiens and Mus musculus Data Availability Online

Since 1998, novel *Homo sapiens* and *Mus musculus* genes and alleles have been announced in ‘IMGT^®^ Creations and updates’ and validated by the IUIS NOM IMGT-NC [114]. Since 2003, IMGT/GENE-DB provides direct links (access from the Query page) which allow the most frequent requests to be encoded in the form of URL: (i) for a given gene {(1). IMGT/GENE-DB entry, (2). IMGT/GENE-DB reference sequences of alleles of a given gene in FASTA format, (3). IMGT/LIGM-DB label sequences in FASTA format, (4). Tables of known IMGT/LIGM-DB cDNA or rearranged gDNA sequences or known IMGT/3Dstructure-DB entries}, or (ii) for genes of a group {(1). IMGT/GENE-DB reference sequences of genes of a group in FASTA format, (2). IMGT/LIGM-DB label sequences in FASTA format}. There are also direct links to IMGT/GENE-DB and generalist genomic databases entries in two formats, HTML tables and CSV format. 

On 25 November 2021, IMGT/GENE-DB data include 732 *Homo sapiens* IG and TR genes (with links to HGNC, NCBI Gene, Ensembl, GenAtlas, GeneCards and UniProt) and 916 *Mus musculus* IG and TR genes (with links to MGI and NCBI Gene). The information, for each IMGT/GENE-DB entry, include: IMGT gene functionality, IMGT gene definition (for *Homo sapiens* and *Mus musculus* IG and TR), the HGNC gene definition (identical to the IMGT gene definition), number of alleles, chromosomal localization and IMGT/LIGM-DB reference sequence(s) for allele *01. IMGT/GENE-DB is updated weekly, with downloads available in different formats, in the “IMGT downloads” section.

## 3. IUIS NOM IMGT-NC Reports for New IG and TR Loci Gene and Allele Names

With the increase in genome sequencing and assembly, the starting point for IG and TR gene identification, description and classification has moved from individual sequences (researchers’ submission to generalist databases) to the IG and TR locus identification in NCBI Whole Genome Assemblies (WGS) (submitted by sequencing groups and analyzed by researchers). 

In order to allow researchers to go ahead with expression studies and to publish their data with IMGT gene names even if the loci are not yet been annotated in IMGT^®^ or in other specialist databases, the IUIS NOM Sub-Committee [114] has created the IUIS NOM IMGT-NC Reports. That initiative allows scientists to propose IMGT gene names for new IG and TR variable (V), diversity (D), joining (J) and constant (C) genes and alleles, for a given locus of a given species, to the IUIS Sub-Committee for approval, based on the IMGT Scientific chart rules and the IMGT-ONTOLOGY concepts of classification (CLASSIFICATION axiom). 

**Figure 2:**
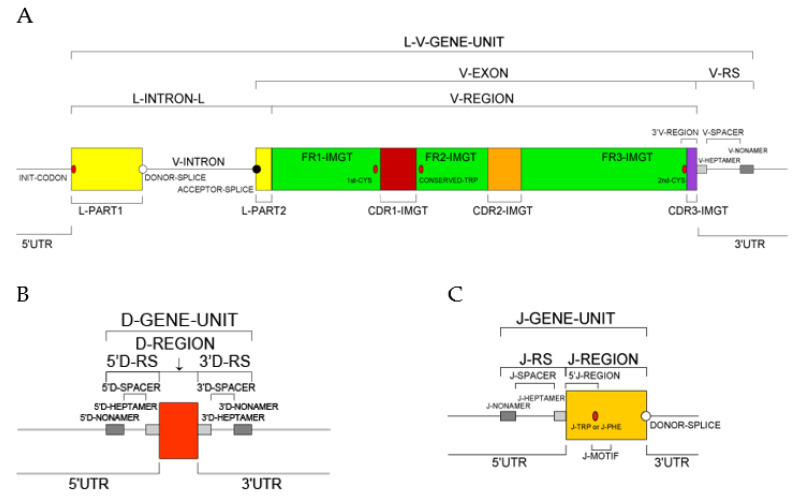
Prototypes with IMGT standardized labels. (**A**) L-V-GENE-UNIT. This label describes gDNA of an IG or TR V-GENE unit, in germline configuration, that comprises L-PART1, V-INTRON, V-EXON and V-RS. (**B**) D-GENE-UNIT. This label describes gDNA of an IG or TR D-GENE unit, in germline configuration, that comprises 5′D-RS, D-REGION and 3′D-RS. (**C**) J-GENE-UNIT. This label describes gDNA of an IG or TR J-GENE unit, in germline configuration, that comprises 5′J-RS and J-REGION. Definitions of the IMGT standardized labels are available at https://www.imgt.org/ligmdb/label# (accessed on 22 February 2022). Abbreviations: L: leader, RS: recombination signal (With permission from M-P. Lefranc and G. Lefranc, LIGM, Founders and Authors of IMGT^®^, the international ImMunoGeneTics information system^®^).

The submission for an IUIS NOM IMGT-NC Report requires that each gene sequence has an accession number in a generalist database (with localization if large original sequence) and that each V, D, J or C gene sequence has been mapped (cloned from bacterial artificial chromosome (BAC), fosmid, cosmid or phage, or extracted from a referenced genome assembly) (Figure 2).

1. For a new V gene and allele, the submitted sequence is that of the L-V-GENE-UNIT: a complete germline genomic sequence (germline gDNA) from the atg (INIT-CODON) of L-PART1 to the V-RS included (http://www.imgt.org/IMGTScientificChart/SequenceDescription/displayimage.php?id=19) (accessed on 22 February 2022) (Figure 2A).
2. For a new D gene and allele, the submitted sequence is that of the D-GENE-UNIT: a complete germline genomic sequence (germline gDNA) from the 5′D-RS to the 3′D-RS included (http://www.imgt.org/IMGTScientificChart/SequenceDescription/displayimage.php?id=2) (accessed on 22 February 2022) (Figure 2B).
3. For a new J gene and allele, the submitted sequence is that of the J-GENE-UNIT plus DONOR-SPLICE: a complete germline genomic sequence (germline gDNA) from the J-RS to the DONOR-SPLICE included (http://www.imgt.org/IMGTScientificChart/SequenceDescription/displayimage.php?id=9) (accessed on 22 February 2022) (Figure 2C).
4. For a new C gene and allele, the submitted sequence is that of the C-GENE-UNIT: a complete genomic sequence (gDNA) from the first codon of the first exon (EX1) to the STOP-CODON included (this requirement has become effective from 1 January 2018), plus the individual exons, if several (http://www.imgt.org/IMGTScientificChart/SequenceDescription/displayimage.php?id=6) (accessed on 22 February 2022). The label C-GENE-UNIT describes gDNA of an IG or TR C-GENE unit, in undefined configuration, that comprises exon(s) (EXON), coding the C-REGION, and intron(s) (INTRON) if present, from the first nucleotide of the first exon to the STOP-CODON (included) after the last exon.

Recent examples of veterinary IG and TR loci from genome assemblies, analyzed by scientists using gene and allele names validated by the IUIS NOM IMGT-NC [114], include: dog (*Canis lupus familiaris*) [134], the first veterinary species with the seven loci identified, cat (*Felis catus*) TR loci [135], rabbit (*Oryctolagus cuniculus*) TRA locus [136], dolphin (*Tursiops truncatus*) [137], Salmonid including salmon (*Salmo salar*) and trout (*Oncorhynchus mykiss*) IGH duplicated loci [138,139] and TRA/TRD locus [140]. These examples of different species and loci have been key elements in the setting of the submission criteria and steps of the now well established IUIS NOM IMGT-NC Reports [114]. They also confirm the necessity for databases using these data (for analysis or biocuration) to cite and link to the original IUIS report to guarantee interoperability. This is illustrated by the links made to the IUIS reports, in IMGT^®^ Creations and updates (http://www.imgt.org/IMGTinformation/creations/) (accessed on 20 February 2022), following data annotation by the IMGT biocurators for data entry in IMGT^®^, as described in Section 6.

## 4. IMGT New Concepts for IG and TR Loci of Jawed Vertebrates Genome Assemblies

### 4.1. Locus in Genome Assembly

**Figure 3:**
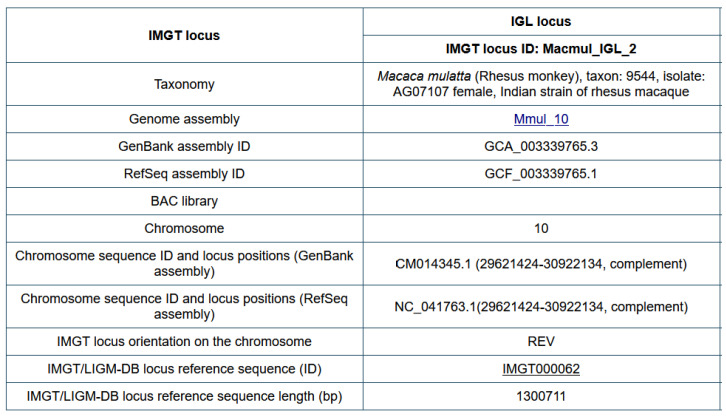
Locus in genome assembly: Rhesus monkey (*Macaca mulatta*) IGL IMGT^®^ http://www.imgt.org (accessed on 20 February 2022), IMGT Web resources > IMGT Repertoire (IG and TR) 1. Locus and genes. >3. Locus descriptions > Locus in genome assembly > IGL: Rhesus monkey http://www.imgt.org/IMGTrepertoire/index.php?section=LocusGenes&repertoire=locusAssembly&species=rhesus_monkey&group=IGL (accessed on 20 February 2022) Only the last annotated locus in genome assembly is shown in the figure, annotated loci of previous assemblies are available online on the right of the displayed locus. (With permission from M-P. Lefranc and G. Lefranc, LIGM, Founders and Authors of IMGT^®^, the international ImMunoGeneTics information system^®^, http://www.imgt.org) (accessed on 20 February 2022).

Before starting IMGT biocuration of a new IG or TR locus of a veterinary species, information is collected in ‘Locus in genome assembly’ (Figure 3). For an easier comparison between loci of different species, and/or between loci of different genomes assemblies (or of different haplotypes, including CNV), the IDENTIFICATION axiom has been enriched by the implementation of ‘IMGT locus ID’ and ‘IMGT/LIGM-DB locus reference sequence (ID)’ (Figure 3).

### 4.2. IMGT Locus ID and IMGT/LIGM-DB Locus Reference Sequence

An ‘IMGT locus ID’ comprises the 6-letter (or 9-letter) code from the genus and species (or subspecies) Latin names (IMGT taxon abbreviations), the locus type and a chronological increasing number, separated by underscores, for example, Macmul_IGL_2 (Figure 3).

An ‘IMGT/LIGM-DB locus reference sequence’ is an IMGT accession number (‘IMGT’ followed by 6 digits) which identifies the IMGT/LIGM-DB flat files containing an IG or TR locus (or part of it) extracted from an NCBI genome assembly and presented in its own 5′ to 3′ locus orientation. As a locus may have, on the chromosome, a forward (or ‘Watson’) (FWD) or a reverse (REV) orientation (IMGT^®^ http://www.imgt.org (accessed on 20 February 2022), IMGT Web resources > IMGT Index > Genomic orientation), the sequence orientation in the IMGT accession number flat file is either unchanged (direct) relative to the sequence on the chromosome for an FWD locus, or reverse complemented for a REV locus. For example, the rhesus macaque (*Macaca mulatta*) IGL locus orientation on chromosome 10 is reverse (REV) and the IMGT/LIGM-DB locus reference sequence in IMGT000062 is reverse-complement relative to the sequence on chromosome 10.

The information from ‘Locus in genome assembly’ (Figure 3) is reported in the definition lines (DE) of the IMGT/LIGM-DB locus reference accession number. For IMGT000062 it includes: *Macaca mulatta* (Rhesus monkey), taxon:9544, isolate: AG07107 single Indian origin rhesus female, assembly Mmul_10, 2345051 [UID], GenBank assembly ID: GCA_003339765.3, Refseq assembly ID: GCF_003339765.1, chromosome 10: CM014345.1 (29621424-30922134, complement), IMGT locus ID: Macmul_IGL_2.

### 4.3. IMGT LOCUS-UNIT Label and Qualifiers

The label IMGT-LOCUS-UNIT (DESCRIPTION axiom) was created to describe a locus, isolated from a genome assembly, in an IMGT accession number flat file. The definition of the IMGT-LOCUS-UNIT and its qualifiers are given in Table 1.

Three IMGT/LIGM-DB locus reference sequences for the *Macaca mulatta* (rhesus monkey) IGH (IMGT000064), IGL (IMGT000062) and IGK (IMGT000063) have recently been created and annotated for the Mmul_10 assembly (GCF_003339765.1). 

As an example, the IMGT000062 qualifiers for the IMGT-LOCUS-UNIT (1..1300711) of the Macmul_IGL_2 (IMGT_locus_ID) are the following:

FT IMGT-LOCUS-UNIT 1..1300711
FT /IMGT_locus_3prime_borne=“RSPH14”
FT /IMGT_locus_3prime_gene=“IGLC7”
FT /IMGT_locus_5prime_borne=“TOP3B”
FT /IMGT_locus_5prime_gene=“IGLV(IV)-127”
FT /IMGT_locus_ID=”Macmul_IGL_2”
FT /IMGT_locus_chromosome=”10”
FT /IMGT_locus_length=“1300711 bp”
FT /IMGT_locus_name=“Macaca mulatta IGL”
FT /IMGT_locus_orientation=“reverse (REV)”
FT /IMGT_locus_positions=“CM014345.1
FT (29621424-30922134 complement)”

### 4.4. IMGT Locus 5′ and 3′ Bornes

The IMGT Locus 5′ borne (IMGT_locus_5prime_borne) and the IMGT Locus 3′ borne (IMGT_locus_3prime_borne) (Table 2) are defined for a standardized comparison of the IG and TR locus delimitation across species. The IMGT bornes are genes coding for a protein (other than IG or TR), conserved between species, located upstream of the first gene (for the IMGT 5′ borne) or downstream of the last gene (for the IMGT 3′ borne) of an IG or TR locus (IMGT^®^ http://www.imgt.org (accessed on 20 February 2022), IMGT Web resources > IMGT Repertoire (IG and TR) > 1. Locus and genes > 3. Locus descriptions > Locus bornes: IGH, IGK, IGL, TRA, TRB, TRG, TRD). If IMGT bornes are not yet identified or are too distant to be included in the locus sequence, a minimal 10 kb sequence is added upstream of the first IG or TR gene in 5′ and/or downstream from the last IG or TR gene in 3′. A preliminary overview of the locus IG and TR 5′ and 3′ bornes is shown in Table 2.

### 4.5. IMGT/GENE-DB Localization in Genome Assemblies

**Figure 4:**
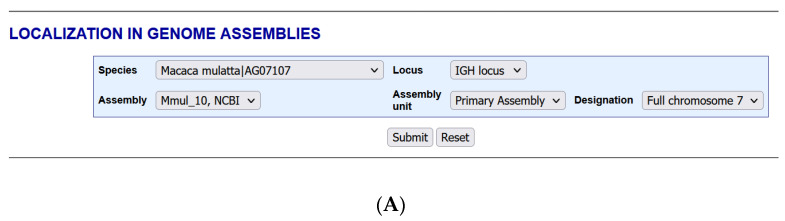

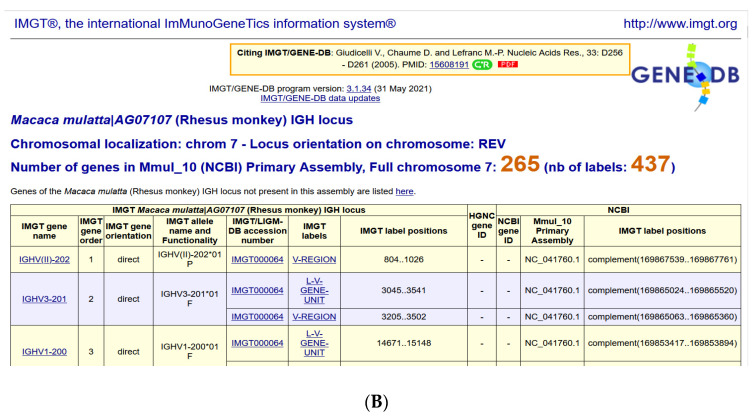
IMGT/GENE-DB Localization in genome assemblies. (**A**) Query for Macaca mulatta|AG07107 (Species, AG07107 isolate) and IGH (Locus) showing the availability of IMGT/GENE-DB biocurated genes for the assembly ‘Mmul_10, NCBI’. (**B**) Top of the results page for the query. IMGT alleles of a given gene are defined by the number which follows the asterisk (i.e., *01) (With permission from M–P. Lefranc and G. Lefranc, LIGM, Founders and Authors of IMGT^®^, the international ImMunoGeneTics information system^®^, http://www.imgt.org) (accessed on 20 February 2022).

The section “LOCALIZATION IN GENOME ASSEMBLIES” (Figure 4) integrated in 2015 in IMGT/GENE-DB, allows, for a given species and a given locus, to query the IMGT IG or TR genes of a given genome assembly. The query Species: Macaca mulatta|AG07107 (AG07107 is the isolate) and Locus: IGH locus shows the availability of IMGT/GENE-DB biocurated genes for the assembly ‘Mmul_10, NCBI’, ‘Primary Assembly’ ‘Full chromosome 7′ (Figure 4A).

The results page for the query Species: Macaca mulatta|AG07107 (Rhesus monkey) Locus: IGH (Figure 4B) provides at the top the chromosome localization: ‘chrom 7′, the locus orientation on chromosome ‘REV’, the number of genes in Mmul_10 (NCBI) (IMGT/GENE-DB annotated genes) ‘265′ and the number of labels ‘437′. For each gene, the information comprises IMGT gene name, IMGT gene order, IMGT gene orientation (direct (5′ > 3′) or opposite (3′ > 5′) in the locus), IMGT allele name and Functionality (F, ORF or P), IMGT/LIGM-DB accession number, IMGT labels (L-V-GENE-UNIT and V-REGION for a V gene, D-GENE-UNIT and D-REGION for a D gene, J-GENE-UNIT and J-REGION for a J gene, C-GENE-UNIT and C-REGION or the different individual exons for a C gene), IMGT label positions in the IMGT/LIGM-DB accession number, HGNC gene ID (for *Homo sapiens*), NCBI gene ID (if available), NCBI Mmul_10 Primary Assembly Chromosome (NC) accession number and IMGT label positions in the NC sequence. 

The list of genes known to belong to the locus but not localized (NL) in the assembly is also provided in this section as this may correspond to polymorphism by copy number variation, insertion/deletion, or gaps in the assembly.

## 5. Immunoglobulin IMGT Copy Number Variations (CNV) and Haplotypes

### 5.1. Homo sapiens IGH Locus

#### 5.1.1. IGH Locus Representation

**Figure 5:**
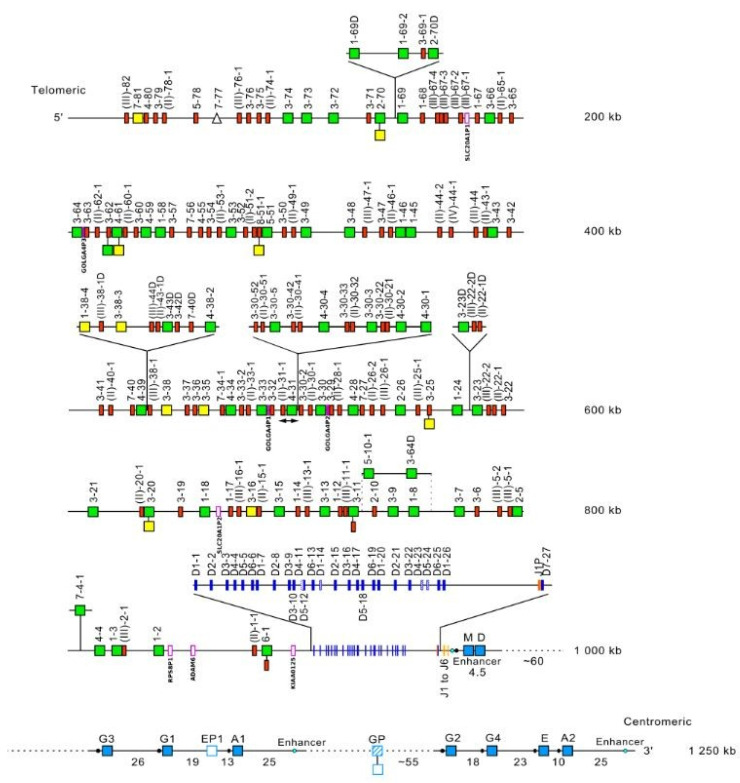
Representation of the human IGH locus at 14q32.33 (REV orientation on the chromosome) [2,5]. The boxes representing the genes are not to scale. Exons are not shown. Switch sequences are represented by a filled circle upstream of the IGHC genes. Pseudogenes that could not be assigned to subgroups with functional genes are designated by a Roman numeral between parentheses, corresponding to the clans, followed by a hyphen, and a number for the localization from 3′ to 5′ in the locus [2]. IMGT^®^ http://www.imgt.org (accessed on 20 February 2022), IMGT Repertoire (IG and TR) 1. Locus and genes > 2. Locus representations > IGH: Human (With permission from M-P. Lefranc and G. Lefranc, LIGM, Founders and Authors of IMGT^®^, the international ImMunoGeneTics information system^®^, http://www.imgt.org) (accessed on 20 February 2022).

The *Homo sapiens* IGH locus is located on chromosome 14, at the telomeric extremity of the long arm, at band 14q32.33 [2]. The orientation of the locus reverse (REV) on the chromosome has been determined by the analysis of translocations, involving the IGH locus, in leukemia and lymphoma. The *Homo sapiens* IGH locus spans 1250 kb [2,5] (Figure 5). The human IGH locus consists of 123 to 129 IGHV genes depending on the haplotypes, 27 IGHD genes belonging to seven subgroups, nine IGHJ genes and, in the most frequent haplotype, 11 IGHC genes [2,5]. Eighty-two to 89 IGHV genes belong to seven subgroups, whereas 43 pseudogenes, which are too divergent to be assigned to subgroups, have been assigned to the clans [2,5] (Figure 5). 

#### 5.1.2. IGH Locus Gene Order, CNV and Haplotypes

1. IGH Locus Gene order

The relative positions (locus gene order) of the IGHV, IGHD, IGHJ and IGHC genes are shown in the *Homo sapiens* IGH locus from its 5′ end to its 3′ end (Table 3). Gene order is according to the IMGT Locus gene order (IMGT^®^ http://www.imgt.org (accessed on 20 February2022 ), IMGT Repertoire (IG and TR) > 1. Locus and Genes > 3. Locus descriptions > Locus gene order > IGH. The number ‘0’ indicates that the relative position is unknown. The three most recently identified genes (IGHV1-68D, IGHV(III-67-4D, IGHV(III-67-3D) are numbered as insertions with a dot (gene order 17.1, 17.2 and 17.3) in the gene order to preserve comparisons with the reference ruler adopted in the description of the *Homo sapiens* IGH polymorphisms [5]. Genes of the related proteins of interest (RPI) used as markers in the locus are indicated with their orientation. 

The gene IGHV(II)-20-1 (gene order 129) is an exception as being only represented by a V-RS. Its V-REGION is replaced by the Alu Homo-sapiens_IGH_Alu-20-1 preceded by an undetermined region and the Alu Homo-sapiens_IGH_Alu-20-3 (AC245166 accession number in IMGT/LIGM-DB).

2. IMGT copy number variation (CNV) nomenclature and definition illustrated with the *Homo sapiens* IGH locus

Copy number variations (CNV) [2,5,141] are numbered from 5′ to 3′ in the locus. (IMGT^®^ http://www.imgt.org (accessed on 20 February 2022), IMGT Web resources > IMGT Repertoire (IG and TR) > Locus gene order > Human (*Homo sapiens*) IGH). Seven CNV are displayed in Table 3, six for the IGHV genes (CNV1 to CNV6) and one (CNV7) for the IGHC genes. The IMGT CNV nomenclature comprises the genus and species (Latin names in italics) (i.e., *Homo sapiens*), the locus (i.e., IGH) and the CNV number (i.e., CNV1). A CNV is delimited by a 5prime gene and a 3prime gene. 

The IMGT standardized definition of a CNV comprises, the group, then between parentheses the order of the start gene (the gene which follows the CNV-5prime), a dash and the order of the end gene (the gene which precedes the CNV-3prime), then the total number of genes involved in the CNV (between the 5prime and 3prime, including RPI gene(s) if present) followed, between parentheses, by the number of IG or TR per functionality (and the number of RPI if present). For instance, the definition of ‘*Homo sapiens* IGH CNV1′ is ‘IGHV(17-20)7(3F,4P)’ (Table 3). The letters ‘i’ for insertion, ‘d’ for deletion, ‘e’ for exchange added to the CNV number indicates the status of a given gene, in a given haplotype for a given CNV, for instance, CNV1i [5] (p 39–40).

3. IMGT CNV haplotypes illustrated with the *Homo sapiens* IGH locus

IMGT CNV haplotypes are described based on the variability of the number of genes present for a given CNV. The description of the CNV is achieved by comparison with the IGH locus [2,5] (IMGT^®^ http://www.imgt.org (accessed on 20 February 2022), IMGT Web resources > IMGT Repertoire (IG and TR) > 1. Locus and genes > 2. Locus representation > IGH: Human). The horizontal main line is conventionally referred to as ‘haplotype A’. 

A well-characterized CNV example is the *Homo sapiens* IGH CNV3 IGHV(87-112)26(8F,16P,2RPI), for which six haplotypes A to F [5,141] correspond to polymorphic amplifications of genes, found in individuals of different populations. These polymorphisms are described as insertion/deletion between IGHV4-34 (86, CNV3-5prime) and IGHV4-28 (113, CNV3-3prime) (Table 4). Haplotype A is from GRCh37 and corresponds to the main line of IMGT Locus Representation [2] (Figure 5). Haplotype B is from GRCh38 and corresponds to BAC clone sequences [141] from the CHORI-17 BAC library. The CNV corresponds to the amplification of a motif of three or four genes: the first one is a pseudogene belonging to the IGHV(II) clan, the second one is a functional gene belonging to the IGHV3 subgroup (in blue) or to the IGHV4 subgroup (in green) and the third one (or the fourth if presence of GOLGA4P1 or GOLGA4P2 (yellow) or IGHV4-30-1) is a pseudogene of the IGHV3 subgroup (Table 4). 

The graphical representation of the CNV3 is available at Human (*Homo sapiens*) Polymorphism by insertion/deletion between IGHV4-34 and IGHV4-28 (haplotypes A to F) on chromosome 14 (14q32.33) (http://www.imgt.org/IMGTrepertoire/index.php?section=LocusGenes&repertoire=locus&species=human&group=IGH/haplotypes#locus) (accessed on 20 February 2022).

**Figure 6:**
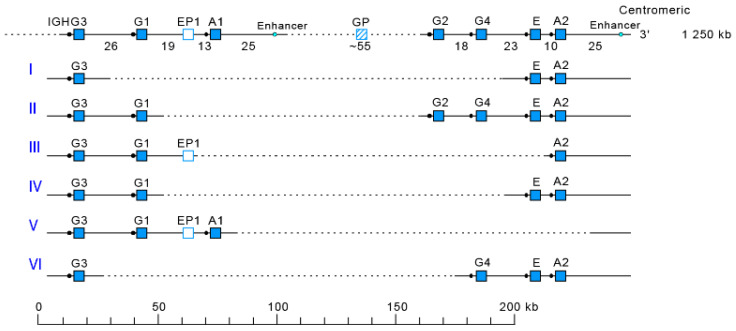
Haplotypes A to G of the *Homo sapiens* IGH CNV7 IGHC(203-211)9(7F,1OP,1P) of the IGH locus on chromosome 14 (14q32.33) [2,5]. The top line corresponds to haplotype A. The multigene deletions I to VI [142,143,144,145,146,147,148] correspond to the CNV7 haplotypes B to G (Table 4). (IMGT^®^ http://www.imgt.org (accessed on 20 February 2022), IMGT Web resources > IMGT Repertoire (IG and TR) > 1. Locus and genes > 2. Locus representation > IGH: > Human IGHC multigene deletions in healthy individuals http://www.imgt.org/IMGTrepertoire/LocusGenes/locus/human/IGH/multigeneIGHC.html (accessed on 20 February 2022). (With permission from M-P. Lefranc and G. Lefranc, LIGM, Founders and Authors of IMGT^®^, the international ImMunoGeneTics information system^®^, http://www.imgt.org) (accessed on 20 February 2022).

The *Homo sapiens* IGH locus on chromosome 14 (14q32.33) is characterized by a remarkable IGH CNV, the CNV7 IGHC(203-211)9(7F,1OP,1P) with seven haplotypes A to G (Table 4), with six of them (haplotypes B to G) corresponding to multigene deletions I to VI (Figure 6), identified on both chromosomes 14 in healthy individuals lacking several subclasses [2,5]. Multigene deletions of haplotypes B to G (either identical or different, on both chromosomes in a given individual) are designated I to VI according to the chronological order in which they were found (reviewed in [5]). Deletion I, first identified by the absence of the Gm1 allotypes in a 70-year-old healthy Tunisian woman (TAK3), homozygous for that deletion [142,143] allowed the ordering of the *Homo sapiens* IGHC genes in the IGH locus [144,145]. Deletions I and II [142,143,146] (haplotypes B and C), found in healthy individuals from consanguineous families, involve highly homologous spots of recombination [147], as also described in a healthy individual (T17) homozygous for deletion III (haplotype D) and lacking IgA1, IgG2, IgG4 and IgE [148]. 

### 5.2. Homo sapiens IGK Locus

#### 5.2.1. IGK Locus Representation

**Figure 7:**
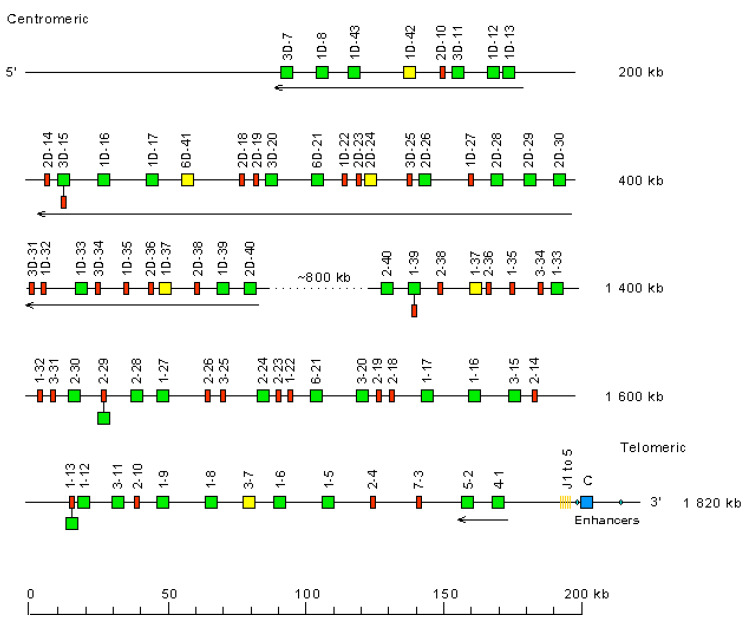
Representation of the human IGK locus at 2p12 (REV orientation on the chromosome) [2,5]. The boxes representing the genes are not to scale. Exons are not shown. The IGKV genes of the proximal V-CLUSTER are designated by a number for the subgroup, followed by a hyphen and a number for the localization from 3′ to 5′ in the locus. The IGKV genes of the distal duplicated V-CLUSTER are designated by the same numbers as the corresponding genes in the proximal V-CLUSTER, with the letter D added. Arrows show the IGKV genes polarity which is opposite to that of the J-C-CLUSTER [2]. IMGT^®^ http://www.imgt.org (accessed on 20 February 2022), IMGT Repertoire (IG and TR) 1. Locus and genes > 2. Locus representations > IGK: Human (With permission from M-P. Lefranc and G. Lefranc, LIGM, Founders and Authors of IMGT^®^, the international ImMunoGeneTics information system^®^, http://www.imgt.org) (accessed on 20 February 2022).

The *Homo sapiens* IGK locus is located on chromosome 2, on the short arm, at band 2p11.2 [2]. The orientation of the locus reverse (REV) on the chromosome has been determined by the analysis of translocations, involving the IGK locus, in leukemia and lymphoma. The *Homo sapiens* IGK locus spans 1820 kb [2,5] (Figure 7). The human IGK locus consists of 76 IGKV genes belonging to seven subgroups, five IGKJ genes and a unique IGKC gene [2,5] (Figure 7). The 76 IGKV genes are organized in two clusters separated by 800 kb [2,5]. The IGKV distal cluster in 5′ of the locus and in the most centromeric position) spans 400 kb and comprises 36 genes. The IGKV proximal cluster (in 3′ of the locus, closer to IGKC, and in the most telomeric position) spans 600 kb and comprises 40 genes [2,5] (Figure 7). 

#### 5.2.2. IGK Locus Gene Order, CNV and Haplotypes

IGK Gene order (Table 5) is according to the IMGT Locus gene order (IMGT^®^ http://www.imgt.org (accessed on 20 February 2022), IMGT Repertoire (IG and TR) > 1. Locus and Genes > 3. Locus descriptions > Locus gene order > IGK) [2,5].

The *Homo sapiens* IGK CNV1 IGKV(1-36)36(16F,4O,14P,1FO,1FP) corresponds to two haplotypes (Table 5). The first one (haplotype A), by far the most common in the populations, is characterized by the presence of the distal cluster in 5′ of the IGK locus and in the most centromeric position (Figure 7). This distal cluster results from the duplication of 36 genes of the proximal cluster and spans 400 kb. The haplotype B lacking the distal cluster has only been found once [2]. 

### 5.3. Homo sapiens IGL Locus

#### 5.3.1. IGL Locus Representation 

**Figure 8:**
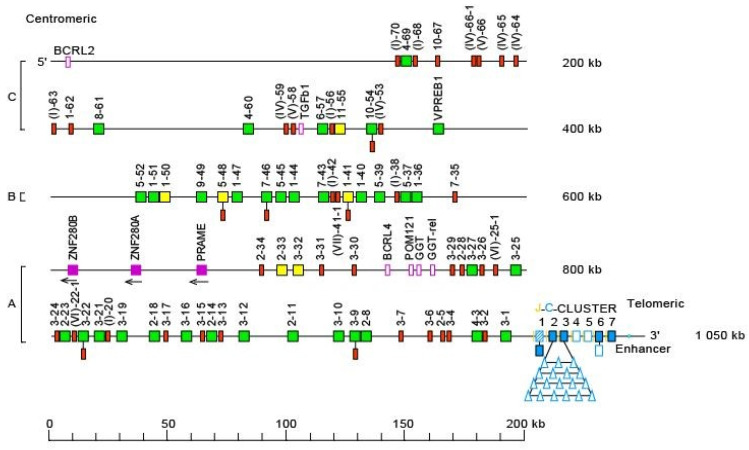
Representation of the human IGL locus at 22q11.2 (FWD orientation on the chromosome) [2,5]. The boxes representing the genes are not to scale. Exons are not shown. (**A**–**C**) refer to three distinct V-CLUSTER based on the IGLV gene subgroup content [149]. Pseudogenes that could not be assigned to subgroups with functional genes are designated by a Roman numeral between parentheses, corresponding to the clans, followed by a hyphen, and a number for the localization from 3′ to 5′ in the locus. IMGT^®^ http://www.imgt.org (Accessed on 20 February 2022), IMGT Repertoire (IG and TR) 1. Locus and genes > 2. Locus representations > IGL: Human (With permission from M-P. Lefranc and G. Lefranc, LIGM, Founders and Authors of IMGT^®^, the international ImMunoGeneTics information system^®^, http://www.imgt.org) (accessed on 20 February 2022).

The *Homo sapiens* IGL locus is located on chromosome 22, on the long arm, at band 22q11.2 [2], The orientation of the locus forward (FWD) on the chromosome has been determined by the analysis of translocations, involving the IGL locus, in leukemia and lymphoma. The *Homo sapiens* IGL locus spans 1050 kb [2,5] (Figure 8). The human IGL locus consists of 73–74 IGLV genes belonging to 11 subgroups, 7 to 11 IGLJ and 7 to 11 IGLC genes depending on the haplotypes, each IGLC gene being preceded by one IGLJ gene [2,5] (Figure 8). The IGLV genes localized on 900 kb define three distinct V-CLUSTER (A, B, C) based on the IGLV gene subgroup content [149] (Figure 8).

#### 5.3.2. IGL Lous Gene Order, CNV and Haplotypes 

IGL gene order is according to the IMGT Locus gene order (IMGT^®^ http://www.imgt.org (accessed on 20 February 2022), IMGT Repertoire (IG and TR) > 1. Locus and Genes > 3. Locus descriptions > Locus gene order > IGL.

One, two, three or four additional IGLC genes, each one most probably preceded by one IGLJ, have been shown to characterize IGLC haplotypes with 8, 9, 10 or 11 genes [150,151] (Table 6). Although these genes have not yet been systematically sequenced, the evidence of the polymorphisms is strongly supported by restriction fragment length polymorphisms (RFLP) and Southern blot analysis [150,151].

## 6. T Cell Receptor IMGT Copy Number Variations (CNV) and Haplotypes 

### 6.1. Homo sapiens TRB Locus

#### 6.1.1. TRB Locus Representation

**Figure 9:**
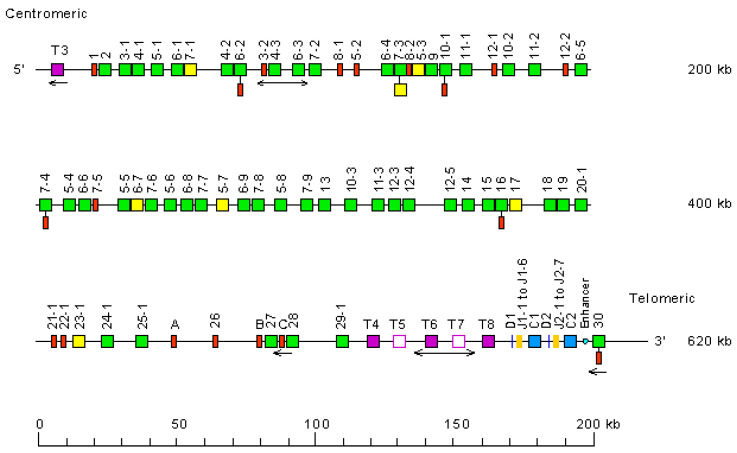
Representation of the human TRB locus at 7q34 (FWD orientation on the chromosome). The boxes representing the genes are not to scale. Exons are not shown. The TRBV genes are designated by a number for the subgroup followed, whenever there are several genes belonging to the same subgroup, by a hyphen and a number for their relative localization in the locus. Numbers increase from 5′ to 3′ in the locus. T3 to T8 indicate trypsinogen or trypsinogen-like genes. T3 (PRSS3P3, TRY3) is at 7.4 kb upstream of TRBV1. Single arrows show genes whose polarity is opposite to that of the D-J-C-CLUSTER. Double arrows indicate insertion/deletion polymorphisms. IMGT^®^ http://www.imgt.org (accessed on 20 February 2022), IMGT Repertoire (IG and TR) 1. Locus and genes > 2. Locus representations > TRB: Human (With permission from M-P. Lefranc and G. Lefranc, LIGM, Founders and Authors of IMGT^®^, the international ImMunoGeneTics information system^®^, http://www.imgt.org) (accessed on 20 February 2022).

The *Homo sapiens* TRB locus is located on chromosome 7, on the long arm, at band 7q34 [3]. The orientation of the locus forward (FWD) on the chromosome has been determined by the analysis of translocations, involving the TRB locus, in leukemia and lymphoma. The *Homo sapiens* TRB locus spans 620 kb [3] (Figure 9). The human TRB locus consists of 64–67 TRBV genes belonging to 32 subgroups. Except for TRBV30, localized downstream of the TRBC2 gene, in the inverted orientation of transcription, all the other TRBV genes are located upstream of a duplicated D-J-C-cluster, which comprises, for the first part TRBD1, six TRBJ and the TRBC1 gene, and for the second part, TRBD2, eight TRBJ and the TRBC2 gene [3] (Figure 9). 

MOXDP2 (monooxygenase DBH-like 2) (5′ borne, opposite orientation relative to the locus) is located upstream of PRSS58 (serine protease 58, trypsinogen-like TRYX3, TRY1) opposite orientation relative to the locus, identified 41 kb upstream of TRBV1 (P). EPHB6 (EPH receptor B6) (3′ borne, direct orientation relative to the locus) has been identified 41 kb downstream of TRBV30 (F), the most 3′ gene in the locus.

#### 6.1.2. TRB Gene Order, CNV and Haplotypes

TRB gene order is according to the IMGT Locus gene order (IMGT^®^ http://www.imgt.org (accessed on 20 February 2020), IMGT Repertoire (IG and TR) > 1. Locus and Genes > 3. Locus descriptions > Locus gene order > TRB. 

A polymorphism by insertion/deletion of 3 genes between the TRBV4-2 and TRBV7-2 genes, encompassing 21 kb, has been described in the human TRB locus [152,153]. It corresponds to haplotype A (L36092) and haplotype B (L36190) and involves three TRBV genes: the pseudogene TRBV3-2, and the functional TRBV4-3 and TRBV6-3 genes [154]. The CNV has been defined as *Homo sapiens* TRB CNV1 TRBV(11-14)4(3F, 1P) (Table 7). A second CNV, *Homo sapiens* TRB CNV2 T4-T8(70-74)5(nr) involves trypsinogene-like genes localized between TRBV29-1 and TRBD1 (Table 6). Two haplotypes have been described, with haplotype B having a deletion of two genes T7 and T8. Detailed sequence analysis of this CNV and characterization of new haplotypes may represent markers of the evolution of the TRB locus between populations and between species.

### 6.2. Homo sapiens TRA/TRD Locus

#### 6.2.1. TRA/TRD Locus Representation

**Figure 10:**
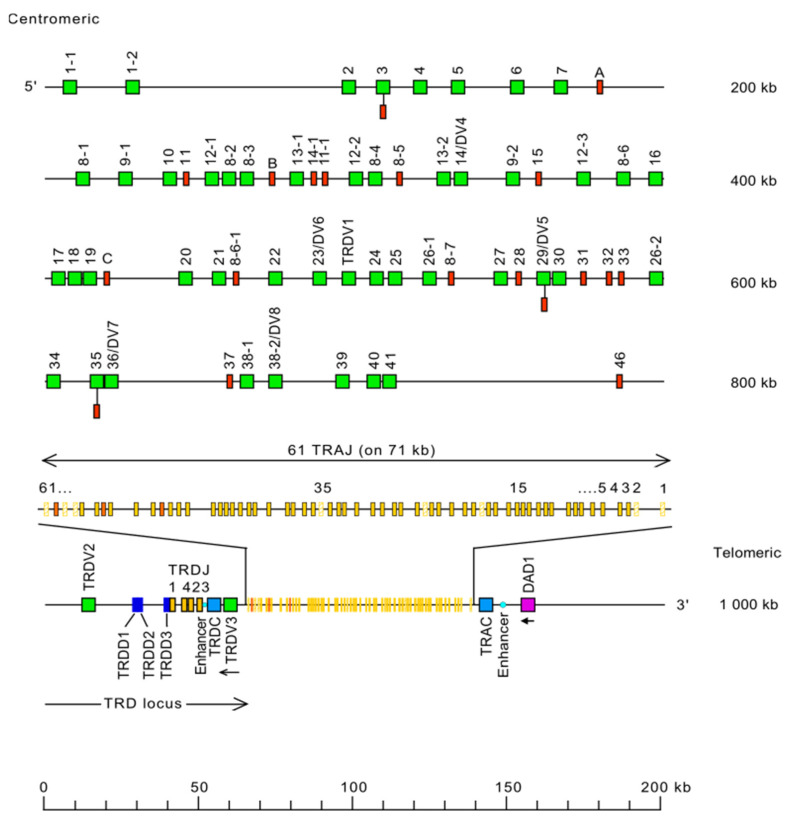
Representation of the human TRA locus at 14q11.2 (FWD orientation on the chromosome) [3]. The boxes representing the genes are not to scale. Exons are not shown. The TRAV genes are designated by a number for the subgroup, followed, whenever there are several genes belonging to the same subgroup, by a hyphen and a number for their relative localization in the locus. Numbers increase from 5′ to 3′ in the locus. The TRD genes are nestled in the TRA locus [3]. IMGT^®^ http://www.imgt.org (accessed on 20 February 2020), IMGT Repertoire (IG and TR) 1. Locus and genes > 2. Locus representations > TRA: Human (With permission from M-P. Lefranc and G. Lefranc, LIGM, Founders and Authors of IMGT^®^, the international ImMunoGeneTics information system^®^, http://www.imgt.org) (accessed on 20 February 2020).

The *Homo sapiens* TRA locus is located on chromosome 14, on the long arm, at band 14q11.2 [3]. The orientation of the locus forward (FWD) on the chromosome has been determined by the analysis of translocations, involving the TRA and TRD loci, in leukemia and lymphoma. The *Homo sapiens* TRA spans 1000 kb [3] (Figure 10). The human TRA locus consists of 54 TRAV genes belonging to 41 subgroups, 61 TRAJ genes localized on 71 kb, and a unique TRAC gene [3] (Figure 10). The organization of the TRAJ genes on a large area is quite unusual and has not been observed in the other IG or TR loci. Moreover, the TRD locus is nestled in the TRA locus between the TRAV and TRAJ genes [3] (Figure 10). V-J rearrangements in the TRA locus, therefore, result in the deletion of the TRD D-J-C cluster genes localized on the same chromosome. This occurs in two steps: first, the deletion of the TRD D-J-C cluster, which results from a rearrangement between deltaRec (sequence located upstream of the cluster) and pseudoJalpha (sequence located downstream of the cluster (this rearrangement generates a T cell receptor excision circle (TREC), a biomarker for normal T cell development), then a TRAV to TRAJ rearrangement.

No 5′ borne conserved between species has been identified upstream of TRAV1-1 (F), the most 5′ gene in the locus. DAD1 (defender against cell death) (3’ borne) has been identified 13 kb downstream of TRAC (F), the most 3’ gene in the locus (Figure 10). 

#### 6.2.2. TRA/TRD Gene Order, CNV and Haplotypes

TRA/TRD gene order is according to the IMGT Locus gene order (IMGT^®^ http://www.imgt.org (accessed on 20 February 2020), IMGT Repertoire (IG and TR) > 1. Locus and Genes > 3. Locus descriptions > Locus gene order > TRA/TRD. 

Although no CNV or haplotype has been described for the *Homo sapiens* TRA/TRD locus, the corresponding columns are available to provide a frame for future descriptions (Table 8).

### 6.3. Homo sapiens TRG Locus

TRG gene order is according to the IMGT Locus gene order (IMGT^®^ http://www.imgt.org (accessed on 20 February 2022), IMGT Repertoire (IG and TR) > 1. Locus and Genes > 3. Locus descriptions > Locus gene order > TRG. 

#### 6.3.1. TRG Locus Representation

**Figure 11:**
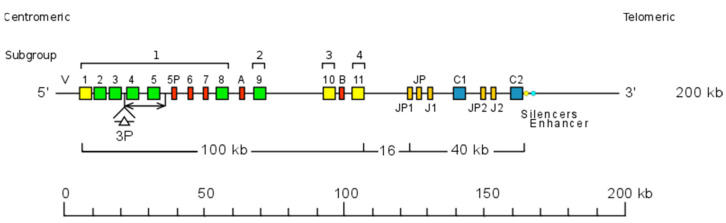
Representation of the human TRG locus at 7p14 (REV orientation on the chromosome) [3]. The boxes representing the genes are not to scale. Exons are not shown. A double arrow indicates an insertion/deletion polymorphism. The TRGV3P gene, a polymorphic gene by insertion has been identified by Southern hybridization in a rare haplotype but has not been sequenced. IMGT^®^ http://www.imgt.org (accessed on 20 February 2022), IMGT Repertoire (IG and TR) 1. Locus and genes > 2. Locus representations > TRG: Human (With permission from M-P. Lefranc and G. Lefranc, LIGM, Founders and Authors of IMGT^®^, the international ImMunoGeneTics information system^®^, http://www.imgt.org) (accessed on 20 February 2022).

The *Homo sapiens* TRG locus is located on chromosome 7, on the short arm, at band 7p14 [3]. The orientation of the locus reverse (REV) on the chromosome has been determined by the analysis of chromosome 7 inversions inv(7)(p14-q34), involving the TRG and TRB loci in ataxia-telangiectasia patients, and in leukemia. The *Homo sapiens* TRG locus spans 160 kb [3,115] (Figure 11). The human TRG locus consists of 12-15 TRGV genes belonging to 6 subgroups, upstream of a duplicated J-C cluster, which comprises, for the first part, three TRGJ and the TRGC1 gene, and for the second part, two TRGJ and the TRGC2 gene [3,115] (Figure 11). TRGV9, expressed in 80–95% of the human peripheral γδ T cells, is the unique member of subgroup 2. TRGV10 and TRGV11, single members of subgroups 3 and 4, respectively, have been found rearranged and transcribed, but they are ORF that cannot be expressed in a gamma chain, due to a splicing defect of the premessenger [3].

AMPH (amphiphysin) (5′ borne) has been identified 16 kb upstream of TRGV1 (ORF), the most 5′ gene in the locus. STARD3NL (STARD3 N-terminal like) (3′ borne) has been identified 9,4 kb downstream of TRGC2 (F), the most 3′ gene in the locus.

#### 6.3.2. TRG Gene Order, CNV and Haplotypes

TRG gene order is according to the IMGT Locus gene order (IMGT^®^ http://www.imgt.org (accessed on 20 February 2022), IMGT Repertoire (IG and TR) > 1. Locus and Genes > 3. Locus descriptions > Locus gene order > TRG. The total number of TRG genes per haploid genome is 19 or 22 of which 11 to 13 are functional (Table 9) [3].

Polymorphisms in the number of TRGV genes and in the exon number of the TRGC2 gene have been described in different populations [155,156,157,158,159,160,161]. A variation of the number of the TRGV subgroup genes (from seven to ten) has been observed [156,157,161]. These allelic polymorphisms, which result from the deletion of V4 and V5, or from the insertion of an additional V gene V3P, between V3 and V4, can be detected by restriction fragment polymorphism (RFLP) [115,156,157,161]. The two TRGC genes, which are 16 kb apart, result, with their associated TRGJ genes, from a recent duplication in the locus. However, there are several structural differences [115]. TRGJP1, TRGJ1, and TRGC1 cross-hybridize to TRGJP2, TRGJ2, and TRGC2, respectively [158,159,160,161], whereas the TRGJP has no equivalent in the duplicated TRGJP2-J2-C2 cluster [160]. The TRGC1 gene has three exons [158], whereas the TRGC2 gene has four or five exons, owing to the duplication of a region that includes exon 2 [155]. The allelic polymorphism of the TRGC2 gene with duplication (C2(2x)) or triplication (C2(3x)) of exon 2 can be identified by RFLP [155]. The exon 2 of the TRGC1 gene has a cysteine [159] involved in the interchain disulfide bridge, whereas this cysteine is not conserved in the exon 2 of the human TRGC2 gene. Enhancer and silencer sequences have been characterized by 6.5 kb downstream of the TRGC2 gene [162].

## 7. IUIS NOM IMGT-NC Validation of IMGT^®^ Creations and Updates

IMGT^®^ Creations and updates (http://www.imgt.org/IMGTinformation/creations/) (accessed on 20 February 2022) are published on the IMGT site after completion of the biocuration of new IG and TR loci, genes and/or alleles. 

The lists of the data available for validation by IUIS NOM IMGT-NC comprise:

1. IMGT/LIGM-DB: the creation of the IMGT Locus reference (i.e., IMGT000062), if relevant, and updates of annotation of previous references sequences.
2. IMGT/GENE-DB: number of genes and alleles per group (IGHV, IGHD, IGHJ and IGHC, or IGKV, IGKJ and IGKC, or IGLV, IGLJ, IGLC), entered in the database, following the annotation of the IMGT Locus reference (with a link to IMGT/GENE-DB update), and FASTA file of the sequences per group.
3. Web resources: creation or update of up to 18 web pages in IMGT Repertoire (IG and TR) (Table 10). These web pages include Locus representation, Locus bornes, Locus description, Locus gene order, Locus in genome assembly, Gene table per group (V, D, J, C), Potential germline repertoire per group (V, D, J), Alignment of alleles per gene (V, D, J, C), Protein displays: per group (V, J, C), and IMGT Colliers de Perles: per gene and domain (V, C), and [CDR1-IMGT.CDR2-IMGT.CDR3-IMGT] lengths: per V subgroup.

The IUIS NOM validation consists in the control of the conformity of the data to the IUIS NOM IMGT-NC requirements for nomenclature assignment and to the IMGT Scientific chart rules based on CLASSIFICATION (genes and alleles names) NUMEROTATION (IMGT unique numbering), DESCRIPTION (labels) and that of their presentation in the IMGT Repertoire (IG and TR) [1,2,3,114].

The seven IG and TR loci of the dog (*Canis lupus familiaris*) and Rhesus monkey (*Macaca mulatta*) have been fully annotated. Species for which most of the IG and TR loci are annotated include cat (*Felis catus*), bovine (*Bos taurus*), sheep (*Ovis aries*) and goat (*Capra hircus*). Standardized IMGT biocuration led to the comparative study of the T cell receptor beta locus of veterinary species [163] based on the *Homo sapiens* TRB locus and to a comparative analysis of *Bos taurus* and *Ovis aries* TRA/TRD loci [164]. A recent comparative study on the evolution of the TRG locus in mammals [165] has highlighted the benefice of using the same IMGT standards, for the same locus, across species. 

If an ‘IMGT^®^ Creations and updates’ correspond to genes previously approved in an ‘IUIS NOM IMGT-NC Report’, a link to the report on the IUIS site is provided [114]. This includes the reports of inferred alleles (new potential alleles deduced by inference from high-throughput sequencing of expressed repertoires) submitted by the “Inferred Allele Review Committee” (IARC) working group of the “Adaptive Immune Receptor Repertoire” (AIRR) community [166].

## 8. Conclusions

IMGT^®^, http://www.imgt.org (accessed on 20 February 2022), the global reference in immunogenetics and immunoinformatics created by Marie-Paule Lefranc (LIGM, Université de Montpellier and CNRS), provides a unique scientific and computing frame for bridging loci, genes, alleles, sequences and structures of the IG or antibodies, TR and MH of the adaptive immune responses in humans and in other jawed vertebrates [167,168,169,170,171,172,173,174,175,176,177,178,179,180,181,182]. The IMGT standards are used for the description of the polymorphisms (genes, allotypes, alleles) [183,184,185,186,187,188,189,190], molecular mechanisms, genome evolution [191,192], susceptibility to diseases, autoimmunity, structure-functions, repertoire in infectious diseases [193,194,195,196,197,198], clonality and sequence analysis in leukemia and lymphoma [199,200,201,202,203,204,205,206], analysis of the content of the Fab and scFv combinatorial phage display libraries screened for identification of novel therapeutic antibody specificities [207,208,209,210,211,212,213], antibody engineering [5,23,26,42,45,47,50,196]. The IMGT locus, gene order, CNV, haplotypes new concepts have been formalized for the comparison of genomic polymorphisms between different assemblies (chromosomes of individuals) in a given species for entries in databases. These concepts are also used for establishing the IMGT nomenclature of the IG and TR genes of new loci of genome assemblies of new species, differences in gene content in haplotypes, from fish to humans, as recently demonstrated, between strains, for the Salmonid IGH and TRA/TRD loci [139,140].

## Figures and Tables

**Table 1:** The IMGT-LOCUS-UNIT label and its associated qualifiers and definitions.

IMGT New Label and Associated Qualifiers		Definition
IMGT label ^a^	IMGT-LOCUS-UNIT	gDNA of an immunoglobulin (IG) or T cell receptor (TR) IMGT locus unit from chromosome genomic assembly, that starts at the 5 prime (5′) end of the most 5′ IG or TR GENE-UNIT in the IMGT-LOCUS-UNIT and ends at the 3 prime (3′) end of the most 3′ IG or TR GENE-UNIT in the locus
IMGT qualifiers ^b^	IMGT_locus_3prime_borne ^c^	Name of the gene identified as the 3 prime (3′) borne of an IMGT-LOCUS-UNIT
	IMGT_locus_3prime_gene	IMGT gene name of the most 3 prime (3′) IG or TR GENE-UNIT of an IMGT-LOCUS-UNIT
	IMGT_locus_5prime_borne ^c^	Name of the gene identified as the 5 prime (5′) borne of an IMGT-LOCUS-UNIT
	IMGT_locus_5prime_gene	IMGT gene name of the most 5 prime (5′) IG or TR GENE-UNIT of an IMGT-LOCUS-UNIT
	IMGT_locus_ID	Identifier of an IMGT-LOCUS-UNIT comprising the IMGT_locus_name and a chronological number, separated with underscores
	IMGT_locus_chromosome	Chromosome identifier (with band or section if known)
	IMGT_locus_length	Length of an IMGT-LOCUS-UNIT in base pairs (bp) in the sequence
	IMGT_locus_name	Name of an IMGT-LOCUS-UNIT, that includes the genus and species Latin names and the IMGT locus type
		(i.e., in higher vertebrates: IGH, IGK, IGL, TRA, TRB, TRG, TRD)
	IMGT_locus_orientation	Orientation of an IMGT-LOCUS-UNIT on a chromosome, is either “forward (FWD)” or “reverse (REV)”
	IMGT_locus_positions	NCBI chromosome sequence accession with positions of the IMGT-LOCUS-UNIT

^a^ IMGT/LIGM-DB labels: http://www.imgt.org/ligmdb/label# (accessed on 20 February 2022). ^b^ IMGT/LIGM-DB qualifiers: http://www.imgt.org/ligmdb/qualifier (accessed on 20 February 2022). ^c^ IMGT Borne: http://www.imgt.org/IMGTindex/IMGTborne.php (accessed on 20 February 2022).

**Table 2:** IMGT Locus 5′ and 3′ bornes of the IG and TR loci.

	IMGT Locus 5′ Borne			IMGT Locus 3′ Borne		
	Gene Name		Occurrence	Gene Name		Occurrence
			/Nb of Species			/Nb of Species
IGH	N.d. ^a,b^		6/6	N.d. ^a,b^		6/6
IGK	PAX8 ^b^	paired box 8	6/10	RPIA ^b^	ribose 5-phosphate isomerase A	10/10
	N.d. ^a^		4/10			
IGL	TOP3B ^b^	DNA topoisomerase III	4/9	RSPH14 ^b^	radial spoke head 14 homolog	5/9
	SLC5A1	solute carrier family 5 member 1	2/9	VPREB3	V-set pre-B cell surrogate light chain 3	3/9
	N.d. ^a^		3/9	N.d. ^a^		1/9
TRA/TRD	OR10G3 ^b^	olfactory receptor 10G3	6/9	DAD1 ^b^	defender against cell death	9/9
	N.d. ^a^		3/9			
TRB	MOXD2 ^b^	monooxygenase DBH-like 2	10/10	EPHB6 ^b^	EPH receptor B6	10/10
TRG	AMPH ^b^	amphiphysin	8/8	STARD3NL	STARD3 N-terminal like	8/8

^a^ N.d.: Not defined; ^b^ *Macaca mulatta*, included in the occurrence/Nb of species.

**Table 3:** *Homo sapiens* IGH locus: IMGT gene order and copy number variations (CNV).

IMGT Gene Name	Functionality	IMGT Gene Order in Locus	IMGT Gene	Copy Number Variations (CNV) Nomenclature, RPI Aliases (If Recent Changes)
			Orientation in Locus	
IGHV1-NL1	P	0	N.d	
IGHV3-NL1	F	0	N.d	
IGHV7-NL1	P	0	N.d	
IGHV(III)-82	P	1	direct	
IGHV7-81	ORF	2	direct	
IGHV4-80	P	3	direct	
IGHV3-79	P	4	direct	
IGHV(II)-78-1	P	5	direct	
IGHV5-78	P	6	direct	
IGHV7-77	N.d.	7	direct	
IGHV(III)-76-1	P	8	direct	
IGHV3-76	P	9	direct	
IGHV3-75	P	10	direct	
IGHV(II)-74-1	P	11	direct	
IGHV3-74	F	12	direct	
IGHV3-73	F	13	direct	
IGHV3-72	F	14	direct	
IGHV3-71	P	15	direct	
IGHV2-70	F, ORF	16	direct	CNV1-5prime
IGHV1-69D	F	17	direct	
IGHV1-68D	P	17.1	direct	
IGHV(III)-67-4D	P	17.2	direct	*Homo sapiens* IGH CNV1
IGHV(III)-67-3D	P	17.3	direct	IGHV(17-20)7(3F,4P)
IGHV1-69-2	F	18	direct	
IGHV3-69-1	P	19	direct	
IGHV2-70D	F	20	direct	
IGHV1-69	F	21	direct	CNV1-3prime
IGHV1-68	P	22	direct	
IGHV(III)-67-4	P	23	direct	
IGHV(III)-67-3	P	24	direct	
IGHV(III)-67-2	P	25	direct	
IGHV(II)-67-1	P	26	direct	
SLC20A1P1	nr	27	direct	RPI (GLVR1)
IGHV1-67	P	28	direct	
IGHV3-66	F	29	direct	
IGHV(II)-65-1	P	30	direct	
IGHV3-65	P	31	direct	
IGHV3-64	F	32	direct	
GOLGA4P3	nr	33	direct	RPI (Golgin)
IGHV3-63	P	34	direct	
IGHV(II)-62-1	P	35	direct	
IGHV3-62	F, P	36	direct	
IGHV4-61	F, ORF	37	direct	
IGHV(II)-60-1	P	38	direct	
IGHV3-60	P	39	direct	
IGHV4-59	F	40	direct	
IGHV1-58	F	41	direct	
IGHV3-57	P	42	direct	
IGHV7-56	P	43	direct	
IGHV4-55	P	44	direct	
IGHV3-54	P	45	direct	
IGHV(II)-53-1	P	46	direct	
IGHV3-53	F	47	direct	
IGHV3-52	P	48	direct	
IGHV(II)-51-2	P	49	direct	
IGHV8-51-1	ORF, P	50	direct	
IGHV5-51	F	51	direct	
IGHV3-50	P	52	direct	
IGHV(II)-49-1	P	53	direct	
IGHV3-49	F	54	direct	
IGHV3-48	F	55	direct	
IGHV(III)-47-1	P	56	direct	
IGHV3-47	P	57	direct	
IGHV(II)-46-1	P	58	direct	
IGHV1-46	F	59	direct	
IGHV1-45	F	60	direct	
IGHV(II)-44-2	P	61	direct	
IGHV(IV)-44-1	P	62	direct	
IGHV(III)-44	P	63	direct	
IGHV(II)-43-1	P	64	direct	
IGHV3-43	F	65	direct	
IGHV3-42	P	66	direct	
IGHV3-41	P	67	direct	
IGHV(II)-40-1	P	68	direct	
IGHV7-40	P	69	direct	
IGHV4-39	F	70	direct	CNV2-5prime
IGHV1-38-4	ORF	71	direct	
IGHV(III)-38-1D	P	72	direct	
IGHV3-38-3	ORF	73	direct	
IGHV(III)-44D	P	74	direct	
IGHV(II)-43-1D	P	75	direct	*Homo sapiens* IGH CNV2
IGHV3-43D	F	76	direct	IGHV(71-80)10(2F,2O,6P)
IGHV3-42D	P	77	direct	
IGHV7-40D	P	78	direct	
IGHV4-38-2	F	79	direct	
IGHV(III)-38-1	P	80	direct	
IGHV3-38	ORF	81	direct	CNV2-3prime
IGHV3-37	P	82	direct	
IGHV3-36	P	83	direct	
IGHV3-35	F, ORF	84	direct	
IGHV7-34-1	P	85	direct	
IGHV4-34	F	86	direct	CNV3-5prime ^a^
IGHV3-33-2	P	87	direct	
IGHV(II)-33-1	P	88	direct	
IGHV3-33	F	89	direct	
GOLGA4P1	nr	90	direct	
IGHV3-32	P	91	direct	
IGHV(II)-31-1	P	92	direct	
IGHV4-31	F	93	direct	
IGHV3-30-52	P	94	direct	
IGHV(II)-30-51	P	95	direct	
IGHV3-30-5	F	96	direct	
IGHV3-30-42	P	97	direct	
IGHV(II)-30-41	P	98	direct	
IGHV4-30-4	F	99	direct	*Homo sapiens* IGH CNV3
IGHV3-30-33	P	100	direct	IGHV(87-112)26(8F,16P,2RPI)
IGHV(II)-30-32	P	101	direct	
IGHV3-30-3	F	102	direct	
IGHV3-30-22	P	103	direct	
IGHV(II)-30-21	P	104	direct	
IGHV4-30-2	F	105	direct	
IGHV4-30-1	F	106	direct	
IGHV3-30-2	P	107	direct	
IGHV(II)-30-1	P	108	direct	
IGHV3-30	F	109	direct	
GOLGA4P2	nr	110	direct	
IGHV3-29	P	111	direct	
IGHV(II)-28-1	P	112	direct	
IGHV4-28	F	113	direct	CNV3-3prime
IGHV7-27	P	114	direct	
IGHV(II)-26-2	P	115	direct	
IGHV(III)-26-1	P	116	direct	
IGHV2-26	F	117	direct	
IGHV(III)-25-1	P	118	direct	
IGHV3-25	ORF, P	119	direct	
IGHV1-24	F	120	direct	CNV4-5prime
IGHV3-23D	F	121	direct	*Homo sapiens* IGH CNV4
IGHV(III)-22-2D	P	122	direct	IGHV(121-123)3(1F,2P)
IGHV(II)-22-1D	P	123	direct	
IGHV3-23	F	124	direct	CNV4-3prime
IGHV(III)-22-2	P	125	direct	
IGHV(II)-22-1	P	126	direct	
IGHV3-22	P	127	direct	
IGHV3-21	F	128	direct	
IGHV(II)-20-1	P	129	direct	
IGHV3-20	F, ORF	130	direct	
IGHV3-19	P	131	direct	
IGHV1-18	F	132	direct	
SLC20A1P2	nr	133	direct	
IGHV1-17	P	134	direct	
IGHV(III)-16-1	P	135	direct	
IGHV3-16	ORF	136	direct	
IGHV(II)-15-1	P	137	direct	
IGHV3-15	F	138	direct	
IGHV1-14	P	139	direct	
IGHV(III)-13-1	P	140	direct	
IGHV3-13	F	141	direct	
IGHV1-12	P	142	direct	
IGHV(III)-11-1	P	143	direct	
IGHV3-11	F, P	144	direct	CNV5-5prime
IGHV2-10	P	145	direct	*Homo sapiens* IGH CNV5
IGHV3-9	F	146	direct	IGHV(145-149)5(4F,1P), split into:
IGHV1-8	F	147	direct	IGHV-e1 (145-147)3(2F,1P)
IGHV5-10-1	F	148	direct	
IGHV3-64D	F	149	direct	IGHV-e2 (148-149)2(2F)
IGHV3-7	F	150	direct	CNV5-3prime
IGHV3-6	P	151	direct	
IGHV(III)-5-2	P	152	direct	
IGHV(III)-5-1	P	153	direct	
IGHV2-5	F	154	direct	CNV6-5prime
IGHV7-4-1	F	155	direct	*Homo sapiens* IGH CNV6 IGHV(155)1(1F)
IGHV4-4	F	156	direct	CNV6-3prime
IGHV1-3	F	157	direct	
IGHV(III)-2-1	P	581	direct	
IGHV1-2	F	159	direct	
RPS8P1	nr	160	direct	
ADAM6	nr	161	direct	
IGHV(II)-1-1	P	162	direct	
IGHV6-1	F, P	163	direct	
FAM30A	nr	164	opposite	RPI (KIAA0125)
IGHD1-1	F	165	direct	
IGHD2-2	F	166	direct	
IGHD3-3	F	167	direct	
IGHD4-4	F	168	direct	
IGHD5-5	F	169	direct	
IGHD6-6	F	170	direct	
IGHD1-7	F	171	direct	
IGHD2-8	F	172	direct	
IGHD3-9	F	173	direct	
IGHD3-10	F	174	direct	
IGHD4-11	ORF	175	direct	
IGHD5-12	F	176	direct	
IGHD6-13	F	177	direct	
IGHD1-14	ORF	178	direct	
IGHD2-15	F	179	direct	
IGHD3-16	F	180	direct	
IGHD4-17	F	181	direct	
IGHD5-18	F	182	direct	
IGHD6-19	F	183	direct	
IGHD1-20	F	184	direct	
IGHD2-21	F	185	direct	
IGHD3-22	F	186	direct	
IGHD4-23	ORF	187	direct	
IGHD5-24	ORF	188	direct	
IGHD6-25	F	189	direct	
IGHD1-26	F	190	direct	
IGHJ1P	P	191	direct	
IGHD7-27	F	192	direct	
IGHJ1	F	193	direct	
IGHJ2	F	194	direct	
IGHJ2P	P	195	direct	
IGHJ3	F	196	direct	
IGHJ4	F	197	direct	
IGHJ5	F	198	direct	
IGHJ3P	P	199	direct	
IGHJ6	F	200	direct	
IGHM	F	201	direct	
IGHD	F	202	direct	CNV7-5prime
IGHG3	F	203	direct	
IGHG1	F	204	direct	*Homo sapiens* IGH CNV7 ^b^
IGHEP1	P	205	direct	IGHC(203-211)9(7F,1OP,1P)
IGHA1	F	206	direct	
IGHGP	ORF, P	207	direct	
IGHG2	F	208	direct	
IGHG4	F	209	direct	
IGHE	F	210	direct	
IGHA2	F	211	direct	

N.d: Not defined (for IG and TR). nr: nonrelevant (for RPI). ^a^ The CNV3-5prime has been moved to IGHV4-34 (F) upstream in the locus (instead of IGHV3-32 (P) in [5]) to select a functional gene as 5prime and include one additional amplification unit, lacking in haplotype F. ^b^ No CNV7-3prime has been defined for the *Homo sapiens* IGH CNV7, owing to the non-identification of a 3′ borne for the IGH locus. CNV7 involving V genes are in pale green (CNV1 to CNV6), CNV7 involving C genes is in pale blue (CNV7).

**Table 4:** *Homo sapiens* IGH locus CNV haplotypes.

CNV.	IGHV Genes	Fct	Gene Order	Haplotypes								
				A	B	C	D	E	F	G		
CNV1-5prime	IGHV2-70	F,ORF	16									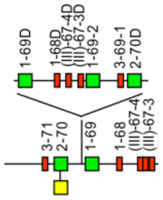
*Homo sapiens* IGH CNV1 IGHV(17-20) 7(3F,4 P)	IGHV1-69D	F	17									
	IGHV1-68D	P	17.1									
	IGHV(III)-67-4D	P	17.2									
	IGHV(III)-67-3D	P	17.3									
	IGHV1-69-2	F	18									
	IGHV3-69-1	P	19									
	IGHV2-70D	F	20									
CNV1-3prime	IGHV1-69	F	21									
CNV2-5prime	IGHV4-39	F	70									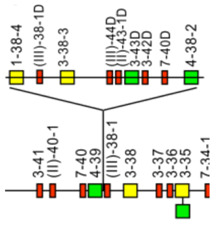
*Homo sapiens* IGH CNV2 IGHV(71-80) 10(2F,2O,6P)	IGHV1-38-4	ORF	71									
	IGHV(III)-38-1D	P	72									
	IGHV3-38-3	ORF	73									
	IGHV(III)-44D	P	74									
	IGHV(III)-43-1D	P	75									
	IGHV3-43D	F	76									
	IGHV3-42D	P	77									
	IGHV7-40D	P	78									
	IGHV4-38-2	F	79									
	IGHV(III)-38-1	P	80									
CNV2-3prime	IGHV3-38	ORF	81									
CNV3-5prime	IGHV4-34	F	86									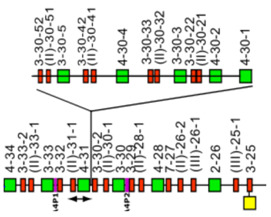
*Homo sapiens* IGH CNV3 IGHV(87-112) 26(8F,16P,2RPI)	IGHV3-33-2	P	87									
	IGHV(II)-33-1	P	88									
	IGHV3-33	F	89									
	GOLGA4P1	nr	90									
	IGHV3-32	P	91									
	IGHV(II)-31-1	P	92									
	IGHV4-31	F	93									
	IGHV3-30-52	P	94									
	IGHV(II)-30-51	P	95									
	IGHV3-30-5	F	96									
	IGHV3-30-42	P	97									
	IGHV(II)-30-41	P	98									
	IGHV4-30-4	F	99									
	IGHV3-30-33	P	100									
	IGHV(II)-30-32	P	101									
	IGHV3-30-3	F	102									
	IGHV3-30-22	P	103									
	IGHV(II)-30-21	P	104									
	IGHV4-30-2	F	105									
	IGHV4-30-1	F	106									
	IGHV3-30-2	P	107									
	IGHV(II)-30-1	P	108									
	IGHV3-30	F	109									
	GOLGA4P2	nr	110									
	IGHV3-29	P	111									
	IGHV(II)-28-1	P	112									
CNV3-3prime	IGHV4-28	F	113									
CNV4-5prime	IGHV1-24	F	120									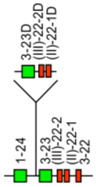
*Homo sapiens* IGH CNV4 IGHV(121-123)3(1F,2P)	IGHV3-23D	F	121									
	IGHV(III)-22-2D	P	122									
	IGHV(II)-22-1D	P	123									
CNV4-3prime	IGHV3-23	F	124									
CNV5-5prime	IGHV3-11	F,P	144									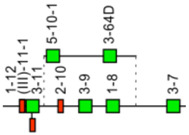
*Homo sapiens* IGH CNV5 IGHV(145-149) 5(4F,1P)	IGHV2-10	P	145									
	IGHV3-9	F	146									
	IGHV1-8	F	147									
	IGHV5-10-1	F	148									
	IGHV3-64D	F	149									
CNV5-3prime	IGHV3-7	F	150									
CNV6-5prime	IGHV2-5	F	154									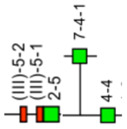
IGHV(155)1(1F) ^a^	IGHV7-4-1	F	155									
CNV6-3prime	IGHV4-4	F	156									
^a^*Homo sapiens* IGH CNV6												
*Homo sapiens* IGH CNV7 IGHC(203-211) 9(7F,1OP,1 P)	IGHG3	F	203									
	IGHG1	F	204									
	IGHEP1	P	205									
	IGHA1	F	206									
	IGHGP	ORF, P	207									
	IGHG2	F	208									
	IGHG4	F	209									
	IGHE	F	210									
	IGHA2	F	211									

Genes present in haplotype A (including CNV-5prime and CNV-3prime) are shown in orange. A pale green color in haplotype A indicates that, in other haplotypes, there is an insertion at these positions. Genes present, as insertion in these other haplotypes by comparison to haplotype A, are shown in green. Genes absent by comparison to haplotype A are shown in red. For CNV3, the column on the right of the haplotypes highlights the duplicated motifs with colors based on the subgroup or clan: blue (IGHV3), green (IGHV4), dark red (IGHV(II). The two golgin genes are in yellow.

**Table 5:** *Homo sapiens* IGK locus: IMGT gene order, copy number variations (CNV) and haplotypes.

IMGT Gene Name	Functionality	IMGT Gene Order in Locus	IMGT Gene Orientation in Locus	Copy Number Variations (CNV)	Haplotypes	
					A	B
IGKV1-NL1	F	0	N.d			
IGKV3D-7	F	1	opposite	*Homo sapiens* IGK CNV1 IGKV(1-36) 36(16F,4O,14P,1FO,1FP)		
IGKV1D-8	F	2	opposite			
IGKV1D-43	F	3	opposite			
IGKV1D-42	ORF	4	opposite			
IGKV2D-10	P	5	opposite			
IGKV3D-11	F	6	opposite			
IGKV1D-12	F	7	opposite			
IGKV1D-13	F	8	opposite			
IGKV2D-14	P	9	opposite			
IGKV3D-15	F, P	10	opposite			
IGKV1D-16	F	11	opposite			
IGKV1D-17	F	12	opposite			
IGKV6D-41	ORF	13	opposite			
IGKV2D-18	P	14	opposite			
IGKV2D-19	P	15	opposite			
IGKV3D-20	F, ORF	16	opposite			
IGKV6D-21	F	17	opposite			
IGKV1D-22	P	18	opposite			
IGKV2D-23	P	19	opposite			
IGKV2D-24	ORF	20	opposite			
IGKV3D-25	P	21	opposite			
IGKV2D-26	F	22	opposite			
IGKV1D-27	P	23	opposite			
IGKV2D-28	F	24	opposite			
IGKV2D-29	F	25	opposite			
IGKV2D-30	F	26	opposite			
IGKV3D-31	P	27	opposite			
IGKV1D-32	P	28	opposite			
IGKV1D-33	F	29	opposite			
IGKV3D-34	P	30	opposite			
IGKV1D-35	P	31	opposite			
IGKV2D-36	P	32	opposite			
IGKV1D-37	ORF	33	opposite			
IGKV2D-38	P	34	opposite			
IGKV1D-39	F	35	opposite			
IGKV2D-40	F	36	opposite			
IGKV2-40	F	37	direct			
IGKV1-39	F, P	38	direct			
IGKV2-38	P	39	direct			
IGKV1-37	ORF	40	direct			
IGKV2-36	P	41	direct			
IGKV1-35	P	42	direct			
IGKV3-34	P	43	direct			
IGKV1-33	F	44	direct			
IGKV1-32	P	45	direct			
IGKV3-31	P	46	direct			
IGKV2-30	F	47	direct			
IGKV2-29	F, P	48	direct			
IGKV2-28	F	49	direct			
IGKV1-27	F	50	direct			
IGKV2-26	P	51	direct			
IGKV3-25	P	52	direct			
IGKV2-24	F	53	direct			
IGKV2-23	P	54	direct			
IGKV1-22	P	55	direct			
IGKV6-21	F	56	direct			
IGKV3-20	F	57	direct			
IGKV2-19	P	58	direct			
IGKV2-18	P	59	direct			
IGKV1-17	F	60	direct			
IGKV1-16	F	61	direct			
IGKV3-15	F	62	direct			
IGKV2-14	P	63	direct			
IGKV1-13	F, P	64	direct			
IGKV1-12	F	65	direct			
IGKV3-11	F	66	direct			
IGKV2-10	P	67	direct			
IGKV1-9	F	68	direct			
IGKV1-8	F	69	direct			
IGKV3-7	ORF	70	direct			
IGKV1-6	F	71	direct			
IGKV1-5	F	72	direct			
IGKV2-4	P	73	direct			
IGKV7-3	P	74	direct			
IGKV5-2	F	75	opposite			
IGKV4-1	F	76	opposite			
IGKJ1	F	77	direct			
IGKJ2	F	78	direct			
IGKJ3	F	79	direct			
IGKJ4	F	80	direct			
IGKJ5	F	81	direct			
IGKC	F	82	direct			

CNV1 involving V genes is in pale green. Genes present in both haplotypes A and B are in orange. The deletion in haplotype B is in red.

**Table 6:** *Homo sapiens* IGL locus: IMGT gene order, copy number variations (CNV) and haplotypes.

IMGT Gene Name	Functionality	IMGT Gene Order in Locus	IMGT Gene Orientation in Locus	Copy Number Variations (CNV), RPI Aliases (if Recent Changes)	Haplotypes				
					A	B	C	D	E
IGLV(I)-70	P	1	direct						
FRAMENP	nr	2	opposite						
IGLV4-69	F	3	direct						
IGLV(I)-68	P	4	direct						
IGLV10-67	P	5	direct						
IGLV(IV)-66-1	P	6	direct						
IGLV(V)-66	P	7	direct						
IGLV(IV)-65	P	8	direct						
IGLV(IV)-64	P	9	direct						
IGLV(I)-63	P	10	direct						
IGLV1-62	P	11	direct						
IGLV8-61	F	12	direct						
IGLV4-60	F, P	13	direct						
IGLV(IV)-59	P	14	direct						
IGLV(V)-58	P	15	direct						
BMP6P1	nr	16	direct						
IGLV6-57	F	17	direct						
IGLV(I)-56	P	18	direct						
IGLV(IV)-55-1	P	18.1	direct						
IGLV11-55	ORF	19	direct						
IGLV10-54	F, P	20	direct						
IGLV(IV)-53	P	21	direct						
VPREB1	F	22	direct	RPI (CD179A)					
IGLV5-52	F	23	direct						
IGLV1-51	F	24	direct						
IGLV1-50	ORF	25	direct						
IGLV9-49	F	26	direct						
IGLV5-48	ORF, P	27	direct						
IGLV1-47	F	28	direct						
IGLV7-46	F, P	29	direct						
IGLV5-45	F	30	direct						
IGLV1-44	F	31	direct						
IGLV7-43	F	32	direct						
IGLV(I)-42	P	33	direct						
IGLV(VII)-41-1	P	34	direct						
IGLV1-41	ORF, P	35	direct						
IGLV1-40	F	36	direct						
IGLV5-39	F	37	direct						
IGLV(I)-38	P	38	direct						
IGLV5-37	F	39	direct						
IGLV1-36	F	40	direct						
IGLV7-35	P	41	direct						
ZNF280B	nr	42	opposite	RPI (5′OY11.1)					
ZNF280A	nr	43	opposite	RPI (3′OY11.1)					
PRAME	nr	44	opposite	RPI (CT130)					
IGLV2-34	P	45	direct						
IGLV2-33	ORF	46	direct						
IGLV3-32	ORF	47	direct						
IGLV3-31	P	48	direct						
IGLV3-30	P	49	direct						
BCRP4	nr	50	direct	RPI (BCRL4)					
POM121L1P	nr	51	opposite	RPI (POM121L1)					
GGTLC2	nr	52	direct	RPI (GGTL4)					
LOC129026	nr	53	direct	RPI (GGTLC1P)					
IGLV3-29	P	54	direct						
IGLV2-28	P	55	direct						
IGLV3-27	F	56	direct						
IGLV3-26	P	57	direct						
IGLV(VI)-25-1	P	58	direct						
IGLV3-25	F	59	direct						
IGLV3-24	P	60	direct						
IGLV2-23	F	61	direct						
IGLV(VI)-22-1	P	62	direct						
IGLV3-22	F, P	63	direct						
IGLV3-21	F	64	direct						
IGLV(I)-20	P	65	direct						
IGLV3-19	F	66	direct						
IGLV2-18	F	67	direct						
IGLV3-17	P	68	direct						
IGLV3-16	F	69	direct						
IGLV3-15	P	70	direct						
IGLV2-14	F	71	direct						
IGLV3-13	P	72	direct						
IGLV3-12	F, P	73	direct						
IGLV(I)-11-1	P	73.1	direct						
IGLV2-11	F	74	direct						
IGLV3-10	F	75	direct						
IGLV3-9	F, P	76	direct						
IGLV2-8	F	77	direct						
IGLV3-7	P	78	direct						
IGLV3-6	P	79	direct						
IGLV2-5	P	80	direct						
IGLV3-4	P	81	direct						
IGLV4-3	F	82	direct						
IGLV3-2	P	83	direct						
IGLV3-1	F	84	direct						
IGLJ1	F	85	direct						
IGLC1	F, ORF	86	direct						
IGLJ2	F	87	direct						
IGLC2	F	88	direct	CNV1-5′prime					
IGLJ2A	N.d	88.1	direct	*Homo sapiens* IGL CNV1 IGLJ-IGLC(88-89)8(N.d)					
IGLC2A	N.d	88.2	direct						
IGLJ2B	N.d	88.3	direct						
IGLC2B	N.d	88.4	direct						
IGLJ2C	N.d	88.5	direct						
IGLC2C	N.d	88.6	direct						
IGLJ2D	N.d	88.7	direct						
IGLC2D	N.d	88.8	direct						
IGLJ3	F	89	direct	CNV1-3′prime					
IGLC3	F	90	direct						
IGLJ4	ORF	91	direct						
IGLC4	P	92	direct						
IGLJCBN5	ORF	93	direct						
IGLC5	P	94	direct						
IGLJ6	F	95	direct						
IGLC6	F, P	96	direct						
IGLJ7	F	97	direct						
IGLC7	F	98	direct						

CNV1 involving J and C genes is in pale blue. In the haplotype representation, CNV-5prime and CNV-3prime as well as the duplicated genes present in the haplotypes B, C, D, E are in orange. A pale orange color indicates that these positions correspond to insertion in other haploypes.

**Table 7:** *Homo sapiens* TRB locus: IMGT gene order, copy number variations (CNV) and haplotypes.

IMGT Gene Name	Functionality	IMGT Gene Order	IMGT Gene Orientation in Locus	Copy Number Variation (CNV)	Haplotypes	
					A	B
TRBV1	P	3	direct			
TRBV2	F	4	direct			
TRBV3-1	F	5	direct			
TRBV4-1	F	6	direct			
TRBV5-1	F	7	direct			
TRBV6-1	F	8	direct			
TRBV7-1	ORF	9	direct			
TRBV4-2	F	10	direct	CNV1-5prime		
TRBV6-2	F	11	direct	*Homo sapiens* TRB CNV1 TRBV(11-14)4(3F,1P)		
TRBV3-2	P	12	direct			
TRBV4-3	F	13	direct			
TRBV6-3	F	14	direct			
TRBV7-2	F	15	direct	CNV1-3prime		
TRBV8-1	P	16	direct			
TRBV5-2	P	17	direct			
TRBV6-4	F	18	direct			
TRBV7-3	F, ORF	19	direct			
TRBV8-2	P	20	direct			
TRBV5-3	ORF	21	direct			
TRBV9	F	22	direct			
TRBV10-1	F, P	23	direct			
TRBV11-1	F	24	direct			
TRBV12-1	P	25	direct			
TRBV10-2	F	26	direct			
TRBV11-2	F	27	direct			
TRBV12-2	P	28	direct			
TRBV6-5	F	29	direct			
TRBV7-4	F, P	30	direct			
TRBV5-4	F	31	direct			
TRBV6-6	F	32	direct			
TRBV7-5	P	33	direct			
TRBV5-5	F	34	direct			
TRBV6-7	ORF	35	direct			
TRBV7-6	F	36	direct			
TRBV5-6	F	37	direct			
TRBV6-8	F	38	direct			
TRBV7-7	F	39	direct			
TRBV5-7	ORF	40	direct			
TRBV6-9	F	41	direct			
TRBV7-8	F	42	direct			
TRBV5-8	F	43	direct			
TRBV7-9	F	44	direct			
TRBV13	F	45	direct			
TRBV10-3	F	46	direct			
TRBV11-3	F	47	direct			
TRBV12-3	F	48	direct			
TRBV12-4	F	49	direct			
TRBV12-5	F	50	direct			
TRBV14	F	51	direct			
TRBV15	F	52	direct			
TRBV16	F, P	53	direct			
TRBV17	ORF	54	direct			
TRBV18	F	55	direct			
TRBV19	F	56	direct			
TRBV20-1	F	57	direct			
TRBV21-1	P	58	direct			
TRBV22-1	P	59	direct			
TRBV23-1	ORF	60	direct			
TRBV24-1	F	61	direct			
TRBV25-1	F	62	direct			
TRBVA	P	63	direct			
TRBV26	P	64	direct			
TRBVB	P	65	direct			
TRBV27	F	66	direct			
TRBVC	P	67	opposite			
TRBV28	F	68	direct			
TRBV29-1	F	69	direct	CNV2-5prime		
T4	nr	70	direct	*Homo sapiens* TRB CNV2 T4-T8(70-74)5(nr)		
T5	nr	71	direct			
T6	nr	72	direct			
T7	nr	73	direct			
T8	nr	74	direct			
TRBD1	F	75	direct	CNV2-3prime		
TRBJ1-1	F	76	direct			
TRBJ1-2	F	77	direct			
TRBJ1-3	F	78	direct			
TRBJ1-4	F	79	direct			
TRBJ1-5	F	80	direct			
TRBJ1-6	F	81	direct			
TRBC1	F	82	direct			
TRBD2	F	83	direct			
TRBJ2-1	F	84	direct			
TRBJ2-2	F	85	direct			
TRBJ2-2P	ORF	86	direct			
TRBJ2-3	F	87	direct			
TRBJ2-4	F	88	direct			
TRBJ2-5	F	89	direct			
TRBJ2-6	F	90	direct			
TRBJ2-7	F	91	direct			
TRBC2	F	92	direct			
TRBV30	F, P	93	opposite			

CNV1 which involve V genes is in pale green. CNV2 which involve trypsinogen-like genes is in violet. Genes present in both haplotypes A and B are in orange. The deletions in haplotypes B are in red.

**Table 8:** *Homo sapiens* TRA/TRD locus: IMGT gene order, copy number variations (CNV) and haplotypes.

IMGT Gene Name	Functionality	IMGT Locus Gene Order in Locus	IMGT Gene Orientation in Locus	Copy Number Variations (CNV)	Haplotypes
TRAV1-1	F	1	direct		
TRAV1-2	F	2	direct		
TRAV2	F	3	direct		
TRAV3	F, P	4	direct		
TRAV4	F	5	direct		
TRAV5	F	6	direct		
TRAV6	F	7	direct		
TRAV7	F	8	direct		
TRAVA	P	9	direct		
TRAV8-1	F	10	direct		
TRAV9-1	F	11	direct		
TRAV10	F	12	direct		
TRAV11	P	13	direct		
TRAV12-1	F	14	direct		
TRAV8-2	F	15	direct		
TRAV8-3	F	16	direct		
TRAVB	P	17	direct		
TRAV13-1	F	18	direct		
TRAV14-1	P	19	direct		
TRAV11-1	P	20	direct		
TRAV12-2	F	21	direct		
TRAV8-4	F	22	direct		
TRAV8-5	P	23	direct		
TRAV13-2	F	24	direct		
TRAV14/DV4	F	25	direct		
TRAV9-2	F	26	direct		
TRAV15	P	27	direct		
TRAV12-3	F	28	direct		
TRAV8-6	F	29	direct		
TRAV16	F	30	direct		
TRAV17	F	31	direct		
TRAV18	F	32	direct		
TRAV19	F	33	direct		
TRAVC	P	34	direct		
TRAV20	F	35	direct		
TRAV21	F	36	direct		
TRAV8-6-1	P	37	direct		
TRAV22	F	38	direct		
TRAV23/DV6	F	39	direct		
TRDV1	F	40	direct		
TRAV24	F	41	direct		
TRAV25	F	42	direct		
TRAV26-1	F	43	direct		
TRAV8-7	P	44	direct		
TRAV27	F	45	direct		
TRAV28	P	46	direct		
TRAV29/DV5	F, P	47	direct		
TRAV30	F	48	direct		
TRAV31	P	49	direct		
TRAV32	P	50	direct		
TRAV33	P	51	direct		
TRAV26-2	F	52	direct		
TRAV34	F	53	direct		
TRAV35	F, P	54	direct		
TRAV36/DV7	F	55	direct		
TRAV37	P	56	direct		
TRAV38-1	F	57	direct		
TRAV38-2/DV8	F	58	direct		
TRAV39	F	59	direct		
TRAV40	F	60	direct		
TRAV41	F	61	direct		
TRAV46	P	62	direct		
TRDV2	F	63	direct		
TRDD1	F	64	direct		
TRDD2	F	65	direct		
TRDD3	F	66	direct		
TRDJ1	F	67	direct		
TRDJ4	F	68	direct		
TRDJ2	F	69	direct		
TRDJ3	F	70	direct		
TRDC	F	71	direct		
TRDV3	F	72	opposite		
TRAJ61	ORF	73	direct		
TRAJ60	P	74	direct		
TRAJ59	ORF	75	direct		
TRAJ58	ORF	76	direct		
TRAJ57	F	77	direct		
TRAJ56	F	78	direct		
TRAJ55	P	79	direct		
TRAJ54	F	80	direct		
TRAJ53	F	81	direct		
TRAJ52	F	82	direct		
TRAJ51	P	83	direct		
TRAJ50	F	84	direct		
TRAJ49	F	85	direct		
TRAJ48	F	86	direct		
TRAJ47	F	87	direct		
TRAJ46	F	88	direct		
TRAJ45	F	89	direct		
TRAJ44	F	90	direct		
TRAJ43	F	91	direct		
TRAJ42	F	92	direct		
TRAJ41	F	93	direct		
TRAJ40	F	94	direct		
TRAJ39	F	95	direct		
TRAJ38	F	96	direct		
TRAJ37	F	97	direct		
TRAJ36	F	98	direct		
TRAJ35	F	99	direct		
TRAJ34	F	100	direct		
TRAJ33	F	101	direct		
TRAJ32	F	102	direct		
TRAJ31	F	103	direct		
TRAJ30	F	104	direct		
TRAJ29	F	105	direct		
TRAJ28	F	106	direct		
TRAJ27	F	107	direct		
TRAJ26	F	108	direct		
TRAJ25	ORF	109	direct		
TRAJ24	F	110	direct		
TRAJ23	F	111	direct		
TRAJ22	F	112	direct		
TRAJ21	F	113	direct		
TRAJ20	F	114	direct		
TRAJ19	ORF	115	direct		
TRAJ18	F	116	direct		
TRAJ17	F	117	direct		
TRAJ16	F	118	direct		
TRAJ15	F	119	direct		
TRAJ14	F	120	direct		
TRAJ13	F	121	direct		
TRAJ12	F	122	direct		
TRAJ11	F	123	direct		
TRAJ10	F	124	direct		
TRAJ9	F	125	direct		
TRAJ8	F, P	126	direct		
TRAJ7	F	127	direct		
TRAJ6	F	128	direct		
TRAJ5	F	129	direct		
TRAJ4	F	130	direct		
TRAJ3	F	131	direct		
TRAJ2	ORF	132	direct		
TRAJ1	ORF	133	direct		
TRAC	F	134	direct		

**Table 9:** *Homo sapiens* TRG locus: IMGT gene order, copy number variations (CNV) and haplotypes.

IMGT Gene Name	Functionality	IMGT Gene Order in Locus	IMGT Gene Orientation in Locus	Copy Number Variations (CNV)	Haplotypes		
					A	B	C
TRGV1	ORF	1	direct				
TRGV2	F	2	direct				
TRGV3	F	3	direct	CNV1-5prime			
TRGV3P	P	4	direct	*Homo sapiens* TRG CNV1 TRGV(4-6)3(2F,1P)			
TRGV4	F	5	direct				
TRGV5	F	6	direct				
TRGV5P	P	7	direct	CNV1-3prime			
TRGV6	P	8	direct				
TRGV7	P	9	direct				
TRGV8	F	10	direct				
TRGVA	P	11	direct				
TRGV9	F	12	direct				
TRGV10	ORF	13	direct				
TRGVB	P	14	direct				
TRGV11	ORF	15	direct				
TRGJP1	F	16	direct				
TRGJP	F	17	direct				
TRGJ1	F	18	direct				
TRGC1	F	19	direct				
TRGJP2	F	20	direct				
TRGJ2	F	21	direct				
TRGC2	F	22	direct				

CNV1 which involves V genes is in pale green. Haplotype B has a deletion of 2 genes (red) compared to haplotype A. Haplotype C has an insertion of 1 gene (green) compared to haplotype A. The pale green color indicates the insertion in other haplotypes.

**Table 10:** IMGT^®^ Creations and updates: List of the web pages validated by IUIS NOM IMGT-NC.

Genus Species	IGH	IGK	IGL	TRB	TRA	TRD	TRG
Locus representation	IGH	IGK	IGL	TRB	TRA/TRD		TRG
Locus bornes	IGH	IGK	IGL	TRB	TRA/TRD		TRG
Locus description	IGH	IGK	IGL	TRB	TRA/TRD		TRG
Locus gene order	IGH	IGK	IGL	TRB	TRA/TRD		TRG
Locus in genome assembly	IGH	IGK	IGL	TRB	TRA/TRD		TRG
Gene table: V	IGHV	IGKV	IGLV	TRBV	TRAV	TRDV	TRGV
Gene table: D	IGHD	nr	nr	TRBD	nr	TRDD	nr
Gene table: J	IGHJ	IGHJ	IGHJ	TRBJ	TRAJ	TRDJ	TRGJ
Gene table: C	IGHC	IGHC	IGHC	TRBC	TRAC	TRDC	TRGC
Potential germline repertoire	IGHV IGHD IGHJ	IGKV	IGLV	TRBV TRBD TRBJ	TRAV	TRDV TRDD TRDJ	TRGV
V, D, J or V, J		nr	nr		nr		nr
		IGKJ	IGLJ		TRAJ		TRGJ
Alignment of alleles: per V	X	X	X	X	X	X	X
Alignment of alleles: per D	X	nr	nr	X	nr	X	nr
Alignment of alleles: per J	X	X	X	X	X	X	X
Alignment of alleles: per C	X	X	X	X	X	X	X
Protein displays: V	IGHV	IGKV	IGLV	TRBV	TRAV	TRDV	TRGV
Protein displays: J	IGHJ	IGKJ	IGLJ	TRBJ	TRAJ	TRDJ	TRGJ
Protein displays: C	IGHC	IGKC	IGLC	TRBC	TRAC	TRDC	TRGC
Colliers de Perles: per V domain	X	X	X	X	X	X	X
Colliers de Perles: per C domain	X	X	X	X	X	X	X
[CDR1-IMGT.CDR2-IMGT.CDR3-IMGT] lengths: per V subgroup	X	X	X	X	X	X	X

## Availability

IMGT^®^ is freely available online for academics and non-profit use at http://www.imgt.org/. All the databases and tools referred to in this article are accessible from IMGT^®^ webpage. The IMGT^®^ software and data are provided to the academic users and NPO’s (Not for Profit Organization(s)) under the CC BY-NC-ND 4.0 license. Any other use of IMGT^®^ material, from the private sector, needs a financial arrangement with CNRS.
